# Policy gaps and food systems optimization: a review of agriculture, environment, and health policies in South Africa

**DOI:** 10.3389/fsufs.2023.867481

**Published:** 2023-08-17

**Authors:** Sithabile Hlahla, Mjabuliseni Ngidi, Sinegugu Evidence Duma, Nafiisa Sobratee-Fajurally, Albert Thembinkosi Modi, Rob Slotow, Tafadzwanashe Mabhaudhi

**Affiliations:** 1 Centre for Transformative Agricultural and Food Systems, School of Agricultural, Earth and Environmental Sciences, College of Agriculture, Engineering and Science, University of KwaZulu-Natal, Pietermaritzburg, South Africa; 2 Future Water Research Institute, Department of Civil Engineering, Faculty of Engineering and the Built Environment, University of Cape Town, Cape Town, South Africa; 3 Department of Agricultural Extension and Rural Resource Management, School of Agricultural, Earth and Environmental Sciences, University of KwaZulu-Natal, Pietermaritzburg, South Africa; 4 School of Nursing and Public Health, College of Health Sciences, University of KwaZulu-Natal, Pietermaritzburg, South Africa; 5 School of Life Sciences, University of KwaZulu-Natal, Pietermaritzburg, South Africa; 6 Department of Genetics, Evolution & Environment, University College, London, United Kingdom; 7 International Water Management Institute, Pretoria, South Africa

**Keywords:** food security, nutrition, co-ordination, transdisciplinary, wicked problem

## Abstract

South Africa faces the triple burden of malnutrition, high poverty levels, unemployment, and inequality. “Wicked problems” such as these require innovative and transdisciplinary responses, multi-stakeholder coordination and collaboration, managing complex synergies and trade-offs, and achieving sustainable outcomes. Through qualitative content analysis of national and provincial sector-based policies, we explored the interlinkages between the agriculture, environment, and health sectors in South Africa in the context of sustainable food and nutrition security and the extent to which these interlinkages are integrated into policy and planning. A systemic analysis of the review outcomes was performed to identify its main learning outcome, the *status quo* in the policy process. The nature of feedback loops was identified, and a leverage point was suggested. The review highlighted that policymakers in the agriculture, environment and health sectors are aware of, and have understood, the relationships among the three sectors. They have also made attempts to address these interlinkages through collaboration and coordination. Unfortunately, this has been met with several challenges due to fragmented sector-specific mandates and targets and a lack of resources for integrated solutions. This creates implementation gaps and unintended duplication of activities, leading to poor service delivery. Transitioning to sustainable and healthy food systems will only be possible after these gaps have been closed and implementation optimization has been achieved. Focusing on meta-level problem-framing, functional collaboration through transdisciplinary approaches, and integrated targets are critical to successful policy implementation and progressive realization of national goals related to sustainable food and nutrition security, unemployment, poverty, and inequality.

## 1 Introduction

South Africa is currently facing a triple burden of malnutrition (Nugent, 2011; Thow et al., 2018). These include rising levels of (i) undernutrition, (ii) diet-related-non-communicable diseases (NCDs) such as Type II diabetes, some forms of cancer, overweight, and obesity, and (iii) micronutrient deficiencies. This significantly impacts vulnerable poor households affected by food and nutrition insecurity, mostly women and children (Mohammadi et al., 2013; Alimoradi et al., 2016; FAO, 2018). A staggering 6.8 million people in the country experience hunger, while an additional 10.4 million lack access to adequate food (Statistics South Africa, 2019b); this translates to ~30% of the population being food insecure. Due to a complex interplay of individual, household, and national factors, this number is projected to increase (FAO, 2011; Statistics South Africa, 2019b). These factors include a growing population; high unemployment, poverty, and inequality rates; economic-slowdown; rapid urbanization and an urban population that prefers water- and energy-dense high-protein and processed diets; inadequate governance structures; climate variability and change (Pereira and Drimie, 2016; Scholtz and Von Bormann, 2016; Statistics South Africa, 2019b); and more recently, the COVID-19 pandemic (Swinnen and McDermott, 2020; UN, 2020).

The COVID-19 health crisis profoundly impacted food and nutrition security in the country as it has disrupted food systems by threatening access to food (HLPE, 2020; Swinnen and McDermott, 2020; UN, 2020). Furthermore, it has resulted in an economic slowdown, nationally and globally (HLPE, 2020), caused a loss of livelihoods and incomes, and threatened the health of the poor and marginalized (IFPRI, 2021). Swinnen and McDermott (2020) concur, adding that the impacts of the pandemic on wellbeing will be large relative to disease mortality rates. In addition, income shocks and lockdowns have changed gender dynamics within households and communities, increasing the disadvantages faced by women (Swinnen and McDermott, 2020). Therefore, the pandemic has pushed the world further behind in reaching the Sustainable Development Goals (SDGs) targets and has exposed the harsh disparities within food systems (IFPRI, 2021). Institutions, regulations, and political processes have an important role to play, directly and indirectly, in developing and implementing solutions to address such challenges (FAO, 2011). These solutions need to include evidence-based policy measures that recognize the complexity and multisectoral and multi-dimensional nature of grand challenges (Nkwana, 2015; Delport, 2019) while de-siloing current policy approaches that address the challenges separately (Prato et al., 2018).

Food and nutrition security spans several sectors, including agriculture, health, and the environment, and a complex crosssectoral and interdisciplinary relationship exists among them (Durojaye and Chilemba, 2018). Food systems can exacerbate food-related health risks. They are also a significant driver of climate change and environmental degradation via their high greenhouse gas emissions and heavy reliance on ecological services (Yu and Wu, 2018). Conversely, climate change impacts can result in crop losses, affecting food and nutrition security, with associated health risks (IPES-Food, 2017; Yu and Wu, 2018). Therefore, the three sectors are deeply intertwined in what is being dubbed the food-health-environment nexus (IPES-Food, 2017). Despite these interlinkages, traditionally, each sector has been governed by its own policies and institutions, explaining why siloed approaches have been adopted to address the challenges associated with food and nutrition security (IPES-Food, 2017; Prato et al., 2018). Coordinating all the efforts of the different departments is often difficult. This has meant that activities, policy discussions and deliberations that impact food and food systems are often fragmented and incoherent (Durojaye and Chilemba, 2018; Prato et al., 2018). However, ignoring these interlinkages and trade-offs can have dire consequences for food systems, society, and sustainable development (Gulati et al., 2013).

According to South Africa’s Constitution (Republic of South Africa, 1996), agriculture, and by extension, food and nutrition security, are functional areas of the national government and its nine provincial governments (Department of Agriculture,1995; Republic of South Africa, 1996; de Visser, 2019). The policy implication is that national and provincial departments develop their policies (Department of Agriculture, 1995). However, because of a constitutionally mandated concurrent competency, the provincial departments are not fully accountable to the national department (Nkwana, 2015). It is notable that the Constitution, in Chapter 3, does guide cooperative governance and Section 146 mechanisms to resolve conflicts in legislation. In response to this mandate, in 1995, the national Department of Agriculture published a White Paper on Agriculture, which aimed to “ensure equitable access to agriculture and promote the contribution of agriculture to the development of all communities, society at large and the national economy, to enhance income, food security, employment, and quality of life in a sustainable manner” (Department of Agriculture, 1995). However, the policy did not take cognizance of the need to engage other sectors to ensure the goal’s attainment, nor did it address the local agriculture governance. This is likely because provincial governments are assumed to have the competency and capacity to translate agricultural governance at the local level. These shortcomings often create a policy-scale mismatch and disharmony, whereby we have a food-secure nation but a growing number of food-insecure households (Pereira and Drimie, 2016).

Against this backdrop, this paper explores the interlinkages among policies within the agriculture, health, and environmental sectors in South Africa, at two administrative levels: the national and provincial level, in the context of food and nutrition security and the extent to which these interlinkages have been integrated into these policies. At the provincial level, the study’s focus was on one of the nine provinces in South Africa, KwaZulu-Natal (KZN), selected because of its high rural population (Statistics South Africa, 2019a) and high levels of unemployment, poverty, food insecurity, and malnutrition (Statistics South Africa, 2019a). We assessed and analyzed the content of policy-related documents published by the national and KZN provincial departments to allow for an understanding of the status quo of the different sectors regarding the food system; establish the level of current knowledge that exists; and provide scope to make suggestions for interlinkages and functional collaborations, if not already available. Lastly, a meta-level archetypal systems view explains the feedback conditions responsible for the current *status quo*.

## 2 Methods

### 2.1 Government structure in South Africa

The government of South Africa consists of three spheres—the national, provincial, and local spheres. The local government structure is further divided into two spheres; one exists in metropolitan areas—the metropolitan municipality, and the other in non-metropolitan areas where a two-tier local government system exists (Hlahla et al., 2019). This system comprises the district municipality and local municipalities (Hlahla et al., 2019). The primary function of the national government is to develop policy frameworks, define norms and standards for service provision, and equitably distribute revenue, while the provincial and local governments implement the majority of public functions and attempt to implement the policies developed at the national level (Momberg et al., 2020). The provincial government exercises legislative and executive powers concurrent with the national government, while the local government has varying degrees of legislative and executive authority (Momberg et al., 2020).

### 2.2 Provincial research setting: KwaZulu-Natal province

KwaZulu-Natal (KZN) is one of nine provinces in South Africa and is located on the country’s eastern coast (KZN PPC, 2011). Located within the province are 11 metropolitan and district municipalities (Hlahla et al., 2019); with a population of 11.3 million, the province has the country’s second-highest population (Statistics South Africa, 2019a). After the Gauteng province, it is the second-largest contributor to South Africa’s gross domestic product (GDP) (KZN EDTEA, 2015). KZN is predominantly rural and comprises a metropolitan municipality, 10 district municipalities and 828 wards (KZN PPC, 2011; KZN Department of Health, 2015). The district municipalities have fifty local municipalities under their jurisdiction (KZN PPC, 2011).

KZN has high levels of poverty, inequality, and unemployment, which indicates high levels of deprivation, negatively impacting human development (KZN PPC, 2016; KwaZulu-Natal Provincial Government, 2017, 2020). Many people in the province cannot access basic and quality services in the health and education sectors, which are necessary to improve their overall quality of life (KZN PPC, 2011, 2016; KwaZulu-Natal Provincial Government, 2017). It is estimated that half of all rural households within KZN live in poverty, and the majority do not have access to acceptable levels of sanitation (KZN PPC, 2011). Also, the poor’s food quality is insufficient to meet the nutritional needs of children (KwaZulu-Natal Provincial Government, 2017). Food insecurity and malnutrition are highest in provinces with large rural populations, and KZN has the highest population of the five predominantly rural provinces (Statistics South Africa, 2019a). The province also has the country’s highest number of social grant beneficiaries, with 4 million recipients, equating to 22.2 % of all grant recipients (KwaZulu-Natal Provincial Government, 2017, 2020).

### 2.3 Data collection and analysis

#### 2.3.1 Assessing cross-sectoral linkages in policy formulation

We conducted a desktop review of policies developed and published by the different departments that have governed the agriculture, health and environment sectors at a national level and within the province of KwaZulu-Natal (KZN) in South Africa to determine whether any explicit links between the three sectors are present. Policy in South Africa can be defined as the government’s stated position on internal or external issues (RSA, 2020). It is usually based on the government’s political priorities, which are noted in the governing party’s manifesto and are part of its programme of action (RSA, 2020). Policy generally contains goals to be pursued and the course of action needed to achieve the goals (RSA, 2020). It provides the written basis for ‘the country’s operations and informs legislation and regulations (RSA, 2020), Policy in the context of this research refers to acts, white papers, regulations, strategies, plans, policies, and amendments gazetted by the South African government, and this served as the inclusion criteria. Policies that were not developed by the agriculture, health and environment sectors at the national and KZN provincial levels were excluded from the study. A total of 210 national policies were identified and reviewed, 41 from the agricultural sector, 60 from the health sector, and 109 from the environmental sector (see Supplementary Tables 1–3). For KwaZulu-Natal, nine policy-related documents were retrieved: two from the agriculture department, four from the health department, and three from the environmental department (Supplementary Tables 4–6).

The policies that govern the different sectors are numerous at a national level and are not always easy to identify and find, even on departmental websites. Hence, to identify and develop a comprehensive list of the documents, the *South Africa Yearbook 2019/2020* was used as an initial guide (https://www.gcis.gov.za/content/resourcecentre/sa-info/yearbook). The Yearbook is an official, authoritative reference on the country, updated annually and provides a comprehensive account of the national government’s programmes and policies (RSA, 2019b). Therefore, it is deemed to be a reliable source of information. Although the programmes and policies listed in the document are considered part of the legislative mandates of the corresponding national departments in the country, the lists are not exhaustive, which is one of the study’s limitations. Therefore, we cross-checked the lists with those provided on each departmental website and other online databases for South African policies, such as the one developed by the University of Cape Town (https://libguides.lib.uct.ac.za/GovtPubs/Policies). Due to the reliance on the Yearbook, there may be possible under-representation of all the policy-related documents. Furthermore, documents that were not the direct mandate (or portfolio) of the departments managing the sector and documents that did not refer to human health were excluded from the list. For the KwaZulu-Natal provincial policies, electronic searches for each department were conducted through the provincial government website and each of the provincial departmental websites.

After retrieval of the documents, the contents were manually searched to examine whether health-, environment-, and agriculture-related factors had been incorporated. Given the importance of land and water to agriculture, the search was expanded to include water and land factors. The extent of this integration was then assessed based on information provided within the documents. The study focused on the information published in the documents, not implementation. Following the assessment of the documents, SWOT analyses were conducted to facilitate an understanding of the strengths and weakness of the national- and KZN provincial governments’ policies with regard to recognizing the interlinkages between agriculture, environment and health in the context of food and nutrition security.

#### 2.3.2 Analysis of the systemic structure of the status quo in the food policy systems of the most vulnerable

A system map, combining causal loop diagramming (CLD) and stock accumulation, demonstrates the systemic structure causing the *status quo*, leading to the mismatch between the goals of the individual provincial departments and the needs of the national policy process. In the current context of this paper, the system of interest refers to the creation of an enabling and equitable agricultural sector that can guarantee food and nutrition security to the most vulnerable section of the population, all of which are aided through efficient and effective policy outcomes.

The efficiency with which protracted problems, such as creating an enabling environment for an adaptive food and nutrition security agenda, can be resolved depends on our ability to comprehend how the real-world conditions (Engels et al., 2019) of public sector governance and policy recommendations interact. Not only is it important to consider how real-world conditions (Engels et al., 2019) impact the goal-seeking policy-making process as system variables, but it is equally essential to understand the underlying systemic structure responsible for causing the current state. In such instances, the influence of feedback loops has to be identified.

In the CLD, arrows show the influence of one variable on another—a change in the cause leads to a change in the effect. The polarity of the arrows indicates the factual relationship between any two nodes, which illustrates the causal link. A simple stock and flow network is also used to depict accumulation and the corresponding rate of change over time. The interplay of feedback loops gives rise to a realistic multi-loop system that explains behavior over time (Morecroft, 2010).

## 3 Results and discussion

This section presents the assessment results and discussion on incorporating agriculture, heath, and the environment within each sector to achieve food security and SWOT analyses of the national and KZN provincial governments regarding their policies. Seventeen of the 210 national policies incorporated factors from the three sectors (Table 1 and Supplementary Table 8), while only one of the nine KZN policies incorporated the sectors (Table 1 and Supplementary Table 8). This is discussed in greater detail in the following sections.

### 3.1 Assessment of policies

#### 3.1.1 National policies

##### 3.1.1.1 Agriculture

Food security is primarily the mandate of South Africa’s agriculture sector. The Republic of South Africa’s Constitution states that access to food is a constitutional right and food security is a national priority (Republic of South Africa, 1996). To fulfill this right, the government has been undertaking several actions, including legislative measures, with many dating back to as early as 1947 for the Fertilizers, Farm Feeds, Agricultural Remedies and Stock Remedies Act, 1947 (Act No. 36 of 1947). However, South Africa’s agricultural sector has undergone numerous social and economic changes since independence in 1994 (OECD, 2006), including reconfiguring national government departments responsible for governing the sector. As of June 2019, the agriculture sector is the mandate of the Department of Agriculture, Land Reform and Rural Development (DALRRD) at the national level (RSA, 2019a). Before this, it was governed by the Department of Agriculture, Forestry and Fisheries (DAFF) (2009–2019) and the Department of Agriculture (1996–2009). In May 2019, the department’s overall goal was to create an “enabling environment for food security and sustainable agrarian transformation and enhancing production, employment and economic growth in the sector” (DAFF, 2015, p. 5).

###### 3.1.1.1.1 Incorporation of health and environmental aspects into national agricultural policies

Of the 41 policy-related documents retrieved and assessed, 28 (68%) incorporate health or environmental aspects or both (Supplementary Table 1). Seven policies (17%) incorporate human health/wellbeing, land, water, and environmental considerations. These are the White Paper on Agriculture (1995); Water Services Act (No. 108 of 1997); the Pesticide Management Policy for South Africa (2010); Integrated growth and development plan (IGDP) for agriculture, forestry and fisheries (2012); the National Policy on Food and Nutrition Security (2014); Agricultural Policy Action Plan (APAP) (2014); and the Draft Climate Smart Agriculture Strategic Framework (2018) (Supplementary Table 1). One policy incorporates land only; three include land and water; three include land and environment; four include land, water and environment; two incorporate water only, and four incorporate the environment only (Supplementary Table 1).

Although more than half of these agriculture-related policies mention health or the environment, most do not acknowledge the cross-linkages between the sectors or discuss the linkages in depth. On the other hand, four documents stand out for the incorporation of other sectors, namely the 1995 White Paper on Agriculture, which emphasizes sustainable agriculture; the Pesticide Management Policy for South Africa, which aims to ensure that pesticide use has minimum impact on human health, the environment, and economic development (DAFF, 2010); the National Policy in Food and Nutrition Security (2014) which aims to ensure the “availability, accessibility and affordability of safe and nutritious food at national and household levels” (DAFF, 2014b, p. 6); and the Draft Climate Smart Agriculture Strategic Framework which aims to develop “effective adaptation responses and increase adaptive capacity to reduce vulnerability and increase the overall resilience of South Africa’s Agriculture, Forestry and Fisheries systems, including their socio-economic and institutional characteristics” (DAFF, 2018, p. 5). The Draft Climate Smart Agriculture Strategic Framework and National Policy in Food and Nutrition Security attempt to address the interlinkages between agriculture, health and the environment in the context of food security. The other policies discuss the impact of climate change and environmental degradation on food production without explicitly mentioning health or human wellbeing. The Framework even calls for a focus on the water-energy-food nexus to “enable and improve the understanding and management of the complex interlinkages between water, energy and food systems (DAFF, 2018). Also, the Framework aims to develop “ effective adaptation responses and increase adaptive capacity to reduce vulnerability and increase the overall resilience of South Africa’s Agriculture, Forestry and Fisheries systems, including their socio-economic and institutional characteristics” (DAFF, 2018, p. 5).

The Pesticide Management Policy for South Africa, which aims to ensure that pesticide use has a minimum impact on human health and the environment (DAFF, 2010), also addresses the agriculture-environment-health nexus. This is evident in its assessment that “human health, environmental quality and economic development depend on effective systems that enable South Africans to manage and use pesticides safely and sustainably” (DAFF, 2010, p. 2).

###### 3.1.1.1.2 Co-ordination of activities and collaboration

The APAP, Pesticide Management Policy for South Africa, National Policy on Food and Nutrition Security, and Draft Climate Smart Agriculture Strategic Framework note the importance of inter-departmental coordination to achieve their goals. The APAP notes that research and innovation are critical to agricultural production and the “fight against joblessness” (DAFF, 2014a, p. 37). The Plan advocates creating partnerships and coordination between government departments, industry and the private sector. DAFF identifies key agencies that it will collaborate with for research and innovation. These agencies include the Agricultural Research Council (ARC), the Department of Science and Innovation (DSI), the Department of Water Affairs and Sanitation (DWS) and the Department of Environment, Forestry and Fisheries (DEFF) [now the Department of Forestry, Fisheries and the Environment (DFFE)].

*The Pesticide Management Policy* states that effective and efficient management of pesticides requires inter-departmental coordination. The Policy adds that the responsibility for the enforcement of pesticide regulation will be shared among the Department of Health, Trade and Industry, Finance (Custom and Excise), Labor, Water Affairs, Environmental Affairs, and the DAFF statutory responsibility (DAFF, 2010).

DAFF and the Department of Social Development developed the *National Policy on Food and Nutrition Security*. It states that the multi-faceted nature of food and nutrition security and its multi-dimensionality mean that it cannot be achieved through a single approach in the form of social relief or agricultural production (DAFF, 2014b). It adds that “food and nutrition security requires well-managed inter-sectoral coordination and the genuine integration of existing policies and programmes in health, education, and environmental protection, as well as in agrarian reform and agricultural development” (DAFF, 2014b, p. 6).

To develop *the Draft Climate Smart Agriculture Strategic Framework*, DAFF consulted a multi-disciplinary team of experts from the Department of Environmental Affairs (DEA), the Department of Rural Development and Land Reform (DRDLR), the Department of Small Business Development (DSBD), farmers, civil society organization (CSOs), private sector, researchers, academia and other development partners (DAFF, 2018). However, noticeably absent from these consultations was the Department of Health. This is a limitation given the nature of the framework and the impact of agriculture and climate change on health. Jackson et al. (2009) note that, ultimately, agricultural policies are health policies, given the strong linkages between food policy and public health. Furthermore, a healthy food system seeks to promote the wellbeing of consumers and farmers, in addition to producers, processors, and distributors (Jackson et al., 2009).

The Draft Climate Smart Agriculture Strategic Framework notes a need to strengthen inter-departmental and intradepartmental coordination on climate-smart agriculture issues. It notes that the lack of coordination results in weak implementation between national, provincial and local governments (DAFF, 2018). The Framework adds that limited inter-departmental and intradepartmental coordination is worsened by the low capacity for cross-sectoral planning related to climate-smart agriculture and ineffective communication between Agriculture, Forestry and Fisheries components and DAFF and other sector departments (DAFF, 2010). DAFF adds that a lack of coordination may result from overlapping mandates of different government institutions (DAFF, 2018).

##### 3.1.1.2 Health

South Africa’s health sector is governed by the Health ministry and the National Department of Health (DoH). The National Health Care Act, 61 of 2003 and the Constitution, are the two legal instruments used by the ministry and the departments. The national government has developed several laws, policies and strategies to govern health and allow for a structured and unified health care system through access, equity, efficiency and sustainability. The Department of Health is also responsible for realizing the second outcome of the 2014–2019 Medium Term Strategic Framework- “a *long and healthy life for all South Africans”* (RSA, 2014, p. 17). Access to health services for all citizens is enshrined in Section 27 (2) of the Constitution. The National Development Plan (NDP) Vision 2030 of 2012 identified specific health-related goals to be achieved by 2030 in South Africa (National Planning Commission, 2012). These goals, directly and indirectly, are related to Sustainable Development Goal (SDG) 2, aiming to end world hunger and ensure global food security by 2030. These include raising the life expectancy of South Africans to at least 70 years; expanding treatment and improving prevention programmes for Human Immunodeficiency Virus (HIV) and Tuberculosis (TB); reducing maternal, infant and child mortality and the reduction of the prevalence of non-communicable diseases (NCDs).

###### 3.1.1.2.1 Incorporation of agriculture and environment into national health policies

The health sector only recently started incorporating environmental and agricultural considerations in 2010, mainly in response to a call from the government in the National Climate Change Response White Paper for all key sectors to participate in efforts to mainstream climate-resilient development in the country (Department of Health, 2014). As a result, less than half of the 60 health-related policies (28%) retrieved and assessed have incorporated agriculture or environmental considerations or both (Supplementary Table 2). Two of the 60 policies incorporate all sectors (agriculture, land, water and environment), including the National Environmental Health Policy (2013) and the *National Climate Change and Health Adaptation Plan 2014-2019*. Four incorporate the environment only, three include agriculture, water and environment, three include agriculture and environment, and another three include land aspects.

However, the policies do not explicitly mention the interlinkages between agriculture, the environment, and health. They refer to how one sector affects the other, for example, how climate change and environmental degradation affect diets. On the other hand, the *Strategic Plan for the Prevention and Control of Non-Communicable Diseases 2013–17* recognizes the interlinkages and mentions increasing concern over the increasing challenges posed by climate change and the loss of biodiversity and their effect on the control and prevention of non-communicable diseases. It emphasizes the need for prompt, robust, coordinated, and multisectoral efforts to address those impacts (Department of Health, 2013b).

###### 3.1.1.2.2 Co-ordination of activities and collaboration

The *National Mental Health Policy Framework and Strategic Plan 2013-2020; National Environmental Health Policy 2013; National Climate Change and Health Adaptation Plan 2014–2019; Republic of South Africa Department of Health Strategic Plan 2015/16–2019/20, National Department of Health Strategic Plan 2010/11–2012/13; Strategic Plan for the Prevention and Control of Non-Communicable Diseases 2013–17; National Department of Health Strategic Plan 2014/15 to 2018/19* note the importance of inter-departmental co-ordination in order the achieve their goals. The *National Mental Health Policy Framework and Strategic Plan 2013–2020* states that DoH facilitates inter-sectoral collaboration to bring together all sectors involved in mental health, including Education, Social Development, Labour, Criminal Justice, Housing, Agriculture and NGOs.

The mission of the *National Environmental Health Policy 2013* is “to improve the health of the environment and the quality of life of all communities through a sustainable, co-ordinated, integrated, comprehensive, and proactive Environmental Health Service at all spheres of government” (Department of Health, 2013a, p. 14). DoH notes that environmental health is a shared responsibility between various government departments, namely, the Department of Water and Sanitation (DWS), DEA, DAFF, Department of Transport (DoT), Department of Mineral Resources (DMR), Department of Labour (DoL), and the Department of Human Settlements (DHS). However, cooperation and collaboration on their various policies, programmes and plans are currently weak and need to be strengthened within these departments. Other agencies play key roles in ensuring the successful implementation of the National Environmental Health policy and delivering health services related to environmental health (Department of Health, 2013a). The DoH adds that “there is a need to formulate multi-faceted cooperative governance structures that will focus on promoting synergies, alignment and harmonization of plans, programmes and policies of these organs of states” (Department of Health, 2013a, p. 25).

*The National Climate Change and Health Adaptation Plan 2014-2019* notes that inter-sectoral cooperation and collaboration, community participation and synergies between climate change adaptation and other public health initiatives are crucial for implementing the Adaptation Plan in the health sector. DoH adds that the development of partnerships and mechanisms of cooperation and collaboration between health and non-health sectors will help South Africa adapt to climate change impacts, and inter-sectoral actions are at the heart of this Plan (Department of Health, 2014).

*The Republic of South Africa Department of Health Strategic Plan 2015/16-2019/20* states that effective inter-sectoral collaboration can help address the social determinants of health and improve health outcomes. The sub-programme aims to collaborate with other government departments, development partners, the private sector and civil society organizations to ensure that weaknesses within the Plan are addressed over the term. It is anticipated that there will be a collaboration with the District and Metropolitan Municipalities, the South African Local Government Association (SALGA), the Department of Cooperative Governance and Traditional Affairs (COGTA), the Department of Environmental Affairs, and the Department of Human Settlements to support the delivery of municipal health services (Department of Health, 2015). To address diet-related obesity, the Plan aimed to collaborate with stakeholders from other government departments, civil society and the food industry to create an enabling environment to curb the prevalence of obesity in 2020 by 10% (Department of Health, 2015).

The *National Department of Health Strategic Plan 2010/11-2012/13* notes that inter-sectoral collaboration within government departments is a key intervention to improve health status education, water and sanitation and housing, and community participation. The Plan adds that “environmental health is a shared responsibility between various government departments in South Africa” (Department of Health, 2013a, p. 25). These departments that were identified include the Department of Water (DWA), Department of Environmental Affairs (DEA), Department of Agriculture, Forestry and Fisheries (DAFF), Department of Transport (DOT), Department of Mineral Resources (DMR), Department of Labour (DOL), Department of Cooperative Governance Traditional Affairs (COGTA) and Department of Human Settlement (DHS) and the National Treasury.

*The Strategic Plan for the Prevention and Control of Non-Communicable Diseases (NCD) 2013-17* reports that inter-sectoral collaboration is important for NCD prevention and control and increased political leadership. DoH states that “effective prevention necessitates a broad multisectoral approach involving different government departments, civil society organizations, the private sector, media as well as commitment to health and wellness from individuals themselves” (Department of Health, 2013b, p. 7). DoH notes that access to healthy foods requires government interventions by (at least) the Departments of Agriculture, Trade and Industry, Finance, Basic and Higher Education, while addressing obesity through physical activity requires the involvement of Sport and Recreation, Transport, Basic Education, Urban Settlements and Trade and Industry (Department of Health, 2013b). Co-ordination with non-governmental organizations and the private sector is also critical (Department of Health, 2013b).

*“The improved health, life chances and quality of life of a population require a shift away from departments working in isolation, and all key sectors must recognise their role in working toward a healthy population. The complex interaction of the social, environmental and economic determinants of health require that all government departments take health into account. This will result in more efficient government in terms of both improved health and achieving development goals”* (Department of Health, 2013b, p. 35).

The *National Department of Health Strategic Plan 2014/15 to 2018/19* does not mention collaboration and coordination. It merely states that DoH intends to collaborate with various stakeholders to develop an accountability framework for the health sector.

Overall, DoH recognizes that environmental health cannot be addressed in silos and “is a shared responsibility between various government departments in South Africa” (Department of Health, 2013a, p. 25). However, fulfilling its role in the agriculturehealth-environment nexus will be difficult. The Department has already acknowledged that collaboration and cooperation efforts on its policies, programmes and plans are weak. Therefore, the department needs to build its capacity for multi-faceted cooperative governance (by learning from other successful departments), which will allow it to better implement and monitor its policies and enable it to play a more prominent and effective role in the nexus and South Africa’s climate-resilient development.

##### 3.1.1.3 Environment

The environmental sector in South Africa is currently governed by the Department of Forestry, Fisheries and the Environment (DFFE). Before this, it was governed by the Department of Environmental Affairs (2009–2019) and the Department of Environmental Affairs and Tourism from 1994 to 2009. In May 2019, President Cyril Ramaphosa announced a reconfiguration of some departments transferring forestry and fisheries’ functions from the Department of Agriculture, Forestry and Fisheries to the Department of Environmental Affairs, forming the Department of the Environment, Forestry and Fisheries (DEFF) (RSA, 2019a). The mandate for DEFF is to “realize the right of citizens to an environment that is not harmful to their health or wellbeing and to have the environment protected for the benefit of present and future generations” (Republic of South Africa Government Communication Information System, 2017, p. 1).

###### 3.1.1.3.1 Incorporation of health and agricultural aspects into national environmental policies

Of the 109 environment-related documents retrieved and assessed, more than half (64%) of them incorporate agriculture or health policies or both (Supplementary Table 3). This is not surprising as South Africa’s environmental issues management has been closely tied to sustainable development, a discourse that dominated the 1990s. As a result, post-Apartheid environmental governance in South Africa is underpinned by the three pillars of sustainable development. Ten policies incorporate all four factors, and these are the *Draft White Paper on Conservation and Sustainable Use of Biodiversity (1997); White Paper on Environmental Management Policy (1998); the White Paper on Integrated Pollution and Waste Management (2000); the White Paper for Sustainable Coastal Development in South Africa (2000)*; the National Biodiversity Strategy and Action Plan (2005); National Framework for Sustainable Development (2008) *NEM: Integrated Coastal Management Act (2009)*; and *the White Paper on National Climate Change Response (NCCR) (2011);* National Strategy for Sustainable Development and Action Plan (2011-2014) (NSSD 1); and South Africa’s National Biodiversity Framework (2019–2024). One incorporates agriculture only, two incorporate agriculture, health and land, six incorporate agriculture, health and water, one policy incorporates agriculture and land, four incorporate agriculture, land and water, one incorporates agriculture and water, five incorporate health only, one policy incorporates health and land, eleven incorporate health, land and water, five health and water, seven include land only, 10 incorporate land and water and five incorporate water only.

Similar to the agriculture and health policy-related documents, the policies do not explicitly mention how the three sectors are interlinked. Still, they refer to how one sector affects the other, such as how climate change and environmental degradation affect diets or how agriculture transforms land, impacting biodiversity. The DEA, in the *White Paper on NCCR,* has attempted to link the three sectors by stating that the negative impacts of climate change will affect food security and the nutritional status of people within vulnerable communities and undermine their resistance to diseases such as HIV/AIDS and tuberculosis (DEA, 2011). The department discusses the impact of weather on diseases such as cholera and states that South Africa plans to integrate climate change considerations into health sector plans (DEA, 2011).

*“Recognising that the nutritional status of individuals is key to building resilience to environmental health threats, ensure that food security and sound nutritional policies form part of integrated approach to health adaptation strategies”* (DEA, 2011, *p. 19)*

The department also recognizes that climate resilience is necessary for food security, water, health, and land reform. As a result, the NCCR White Paper states that all sectors should take part in fulfilling the government’s vision for an effective response and just transition to a climate-resilient and low-carbon economy and society (DEA, 2011). These sectors should include water, agriculture, commercial forestry health, biodiversity and ecosystems, and human settlements (DEA, 2011). These key sectors will formulate, implement, publish and regularly update policies, measures, and programmes to mitigate their emission of greenhouse gases (GHGs) and adapt to climate change impacts (DEA, 2011).

South Africa’s National Biodiversity Framework (2019-2024) notes that there is a link between biodiversity and agriculture, stating that while the core work of other sectors such as agriculture, fisheries, water and sanitation, mining, energy, rural development and land reform, urban development, local economic development, and education, is not biodiversity conservation, their businesses impact on the status of biodiversity and ecosystems, or depend on biodiversity assets, ecosystem services and ecological infrastructure (DEFF, 2021). As a result, the Framework states that many decisions affecting biodiversity are taken outside the biodiversity or environmental sector, making it important to adopt a framework for integrated, policy-aligned decision-making (DEFF, 2021).

These policies illustrate that South Africa has successfully developed a progressive and comprehensive legal-institutional environment governance framework (Harsant, 2004; Kotzé, 2006). However, this does not speak to the implementation of the framework, which is beyond this paper’s scope. South Africa is notorious for developing sound policies but is lagging regarding implementation and translating the policies into action on the ground (Tebele, 2016).

###### 3.1.1.3.2 Co-ordination of activities and collaboration

Several environment-related documents highlight the importance of inter-departmental collaboration and coordination in achieving their mandated roles. For example, the *Draft White Paper on Conservation and Sustainable Use of Biodiversity (1997)* states that biodiversity transcends political, institutional and social boundaries. An enabling framework will be provided for the future coordination and cooperation of biodiversity-related activities, plans, programmes and policies in South Africa. The Department of Environmental Affairs and Tourism will collaborate with interested and affected parties to meet its goal of conserving biodiversity and encourages collaboration among the private sector, research institutions, government and non-governmental organizations, and communities to promote the transfer of environmentally sound technologies. The Department adds that the conservation and sustainable use of biodiversity will also be integrated strategically at all levels into national, provincial, local and sectoral planning, programmes, and policy efforts such as forestry, agriculture, fisheries, land reform, industry, education, health, mining to allow for the fulfillment of the goals and objectives of the policy (Department of Environmental Affairs Tourism, 1997).

The Department adds that efforts to conserve biodiversity have been hampered by duplication of efforts, a profusion of laws, a lack of co-ordination, a lack of integration of biodiversity considerations into national decision-making, weak political will concerning environmental conservation, and the insufficient and declining allocation of resources to conservation (Department of Environmental Affairs Tourism, 1997). In addition, it is noted in the *National Biodiversity Strategy and Action Plan* that several national departments in South Africa administer biodiversity-related legislation, while a host of national agencies, provincial departments, and provincial agencies have mandates related to biodiversity conservation and management, creating numerous overlaps (DEAT, 2005). These overlaps often result in confusion and a lack of implementation. Moreover, the Plan states that it is important for municipalities to have the capacity to integrate biodiversity considerations into their spatial and economic planning and environmental management programmes. Many of the issues identified in these policies were identified as early as 1997 and they still exist today, illustrating the failure of the national government to find suitable solutions, decades later.

The *National Biodiversity Strategy and Action Plan* also highlight that the institutional location of state organs is not optimal (DEAT, 2005). For example, museums are placed within the Department of Arts and Culture, raising concerns that the majority of the country’s animal biodiversity collections and the taxonomic research associated with them have become marginalized from mainstream science, leading to a weakening of South Africa’s animal identification, classification and biogeographic services (DEAT, 2005). Therefore, it is necessary to assess various departments and spheres of government mandates and identify any amendments required to ensure a more efficient administration and effective implementation (DEAT, 2005). The *White Paper on Environmental Management (1998)* calls for coordination within and between government departments and agencies to achieve sustainable development. The Department of Environmental Affairs and Tourism adds that environmental concerns affect all aspects of life and must be integrated into all government institutions’ work. “This requires intergovernmental harmonization of policies, legislation, monitoring, regulation and other environmental functions” (DEAT, 1998, p. 21).

The *White Paper on Integrated Pollution and Waste Management (2000)* plans to implement cooperative governance to curb the country’s unacceptably high pollution levels. In the paper, the Department of Environmental Affairs and Tourism adds that current measures to deal with pollution are fragmented and uncoordinated. There are insufficient resources to implement and monitor existing legislation. The Department is planning to eliminate current fragmentation, duplication and lack of co-ordination by reviewing all existing legislation and preparing a single piece of legislation dealing with all waste and pollution matters (DEAT, 2000b).

The *White Paper for Sustainable Coastal Development in South Africa (2000)* calls for coordinating activities to achieve sustainable coastal development and management. “In the past, South African coastal management efforts were fragmented and uncoordinated and were undertaken largely on a sectoral basis. This Policy supports a holistic way of thinking by promoting co-ordinated and integrated coastal management, which views the coast as a system” (DEAT, 2000a, p. 8).

*In the NEM: Biodiversity Act (No. 10 of 2004)*, the *Department of Environmental Affairs and Tourism* calls for co-operative governance in biodiversity planning and seeks to provide for an integrated, co-ordinated and uniform approach to biodiversity management by organs of state in all spheres of government, non-governmental organizations, the private sector, local communities and the public (DEAT, 2004).

The Department of Environmental Affairs, in the *White Paper on National Climate Change Response (2011)*, recognizes that the cross-cutting nature of climate change impacts requires a national policy response that is coordinated, coherent, efficient and effective. The DEA aims to respond to integrate adaptation strategies into the following sectoral plans: the National Water Resource Strategy, as well as reconciliation strategies for particular catchments and water supply systems; the Strategic Plan for South African Agriculture; the National Biodiversity Strategy and Action Plan, as well as provincial biodiversity sector plans and local bioregional plans; the Department of Health Strategic Plan; the Comprehensive Plan for the Development of Sustainable Human Settlements; and the National Framework for Disaster Risk Management.

#### 3.1.2 KwaZulu-Natal provincial policies

##### 3.1.2.1 Agriculture

###### 3.1.2.1.1 Incorporation of health and environmental aspects into KZN agricultural policies

The agriculture sector in KwaZulu-Natal is governed by the KZN Department of Agriculture and Rural Development. KZN has the highest agricultural potential in South Africa, with 17% of the land surface being arable, and 7.5% is high potential (KZN DARD, 2015a). Therefore, the provincial government intends to harness agriculture’s potential to ensure food security and increase its contribution to the provincial economy (KZN DARD, 2015a). This will be achieved by progressing from subsistence food security activities to emerging and commercial farmer development (KZN DARD, 2015a). In line with these goals, the KZN DARD has produced two strategies: the *Strategic Plan 2015–2022* (KZN DARD, 2015a); and the *Strategy for Agrarian Transformation in KZN* (KZN DARD, 2015b). The Strategic Plan has incorporated the environment, and land sectors, while the Strategy for Agrarian Transformation has not incorporated any other sectors. However, the Strategic Plan 2015–2022 does not explicitly mention how the environment and agriculture are interlinked. It merely notes that food security is a complex problem that “transcends social, health and economic boundaries, and, therefore, requires a comprehensive and multi-disciplinary response that will prioritize the eradication of hunger and malnutrition; alleviate poverty and inequality; promote increased access and production of sufficient and diverse food; employment creation and economic growth” (KZN DARD, 2015a). A major focus of the plan is on fulfilling the department’s mandate of promoting integrated rural development over five years by incorporating “elements of economic development, reduction of vulnerability and environmental sustainability whilst building on the inherent strengths of the local people and natural resources”. The plan also notes that optimal land use within commercial farming areas is necessary, which can be achieved via conservation agriculture/climate-smart agriculture and land redistribution (KZN DARD, 2015a).

###### 3.1.2.1.2 Co-ordination of activities and collaboration

Very little reference is made to inter-sectoral coordination of activities. The Strategic Plan calls for a better-co-ordinated approach for efficient and effective service delivery in rural areas and integrated and vibrant rural development (KZN DARD, 2015a).

##### 3.1.2.2 Health

###### 3.1.2.2.1 Incorporation of agriculture and environment into KZN health policies

In KZN, the health sector is governed by the KZN provincial Department of Health (DoH). The government developed four policy documents- *the KwaZulu-Natal Health Act (Act No. 04 of 2000)* (KZN DoH, 2000); the *Health care risk waste management policy for KwaZulu-Natal province* (KZN DoH, 2008), *the KwaZulu-Natal Health Act (Act No. 01 of 2009)* (KZN DoH, 2009); and *the Strategic Plan (2015-2019)* (KZN Department of Health, 2015). All four documents referred to other sectors. The Health Act (No. 04 of 2000) included environmental and water considerations. The Health Act (No. 01 of 2009) and Health care risk waste management policy referred to the environmental sector, and the Strategic Plan incorporated water. The Health Act (No. 04 of 2000) and the Health Act (No. 01 of 2009) focus on environmental health and the function of the environmental health officer, where environmental services are defined as “the anticipation, identification, evaluation, monitoring, promotion and prevention or control of all physical, chemical, biological and aesthetic factors which affect the development, health or wellbeing and survival of a person or community” (KZN DoH, 2000).

The Health Care Risk Waste Management Policy focuses on minimizing the impact of health care risk waste on human health and the environment, from generation to disposal. Regarding water, the Strategic Plan refers to the access to basic services and service delivery of water and sanitation in the province, which are “social determinants of health, along with waste removal and electricity” (KZN EDTEA, 2015).

###### 3.1.2.2.2 Co-ordination of activities and collaboration

Very little reference is made to inter-sectoral coordination of activities. The Health Acts only emphasize the Provincial Health Authority’s need to coordinate the implementation of national and provincial health policies (KZN DoH, 2000, 2009). The Health care risk waste management policy for KwaZulu-Natal notes that the province must establish a provincial waste management committee which must have a representation of senior officials from key components and Departments, including Monitoring and Evaluation, Environmental Health, Quality Assurance, Infection Control, Occupational Health and Safety, Infrastructure Development, Supply Chain Management and Pharmaceutical Services in the Department of Health, Waste Water Quality Management of the Department of Water Affairs and Forestry, Pollution Control and Waste Management of the Department of Agriculture and Environmental Affairs, Relevant NGOs and professional associations (KZN DoH, 2008).

##### 3.1.2.3 Environment

###### 3.1.2.3.1 Incorporation of health and agricultural aspects into KZN environmental policies

The KZN environmental sector is governed by the Department of Economic Development, Tourism and Environmental Affairs (EDTEA). The department developed three policy documents- the *EDTEA Revised Strategic Plan 2014–2019* (KZN EDTEA, 2014), the *EDTEA Revised Strategic Plan 2015–2020* (KZN EDTEA, 2015), and the *KZN Environmental Implementation Plan (EIP)*^1^ (KZN EDTEA, 2016). Two documents referred to other sectors; EDTEA Revised Strategic Plan 2015–2020 incorporated the agricultural sector, while the KZN Environmental Implementation Plan (EIP) incorporated all four sectors. The EDTEA Revised Strategic Plan 2015–2020 highlights KZN’s agriculture sector’s importance to the country (KZN EDTEA, 2015). The EIP identified the policies, plans and programmes within each of the provincial and relevant national departments and local government that could significantly impact the environment and indicated measures that departments are already putting into place to improve their environmental performance and co-operative governance (KZN EDTEA, 2016). These departments include the KZN Departments of Health and Department of Agriculture and Rural Development.

###### 3.1.2.3.2 Co-ordination of activities and collaboration

Very little reference is made to inter-sectoral coordination of activities within the Strategic Plans. Conversely, the Environmental Implementation Plan (EIP) emphasizes coordination and collaboration between sectors. This is because the purpose of the plan is to “co-ordinate and harmonize the environmental policies, plans, programmes and decisions of the various national departments that exercise functions that may affect the environment or are entrusted with powers and duties aimed at the achievement, promotion, and protection of a sustainable environment, and of provincial and local spheres of government, to (i) minimize the duplication of procedures and functions; and (ii) promote consistency in the exercise of functions that may affect the environment” (KZN EDTEA, 2016). The plan seeks to improve the effectiveness and efficiency of service delivery to the public through governance to achieve holistic governance by re-inventing the current fragmented government structures (KZN EDTEA, 2016).

##### 3.1.2.4 Assessment of KZN provincial policies

The agriculture, environmental and health departments within KwaZulu-Natal have developed policies to govern their respective sectors. However, these policies have not done much by integrating other sectors in domains where the sectors influence each other such as food and nutrition security. For example, none of the KZN health sector policies included agricultural considerations. Within the KZN provincial priorities, food security falls under the third priority of human and community development (KZN PPC, 2016). Within this priority, safeguarding and enhancing sustainable livelihoods and food security is strategic objective three (KZN PPC, 2016). The ranking of food security within the provincial priorities may have contributed to the poor integration and co-ordination between the different sectors responsible for food and nutrition security, as well as the lack of incentives. The nature of food and nutrition security is framed and perceived at a political level is important for its realization. While the government is right to frame food security as a human and community development issue, it might be more beneficial for food and nutrition security to be one of the main priorities instead of being part of an objective within a provincial priority.

The KZN Environmental Implementation Plan (EIP), developed by the Department of the Environment, Forestry and Fisheries (DEFF), is the only document that has incorporated all the sectors as it is meant to be utilized as a tool to achieve cooperative environmental governance and sustainable development within the province (KZN EDTEA, 2016). The document is well-written and quite comprehensive in detailing the policies within each of the provincial and national departments and local government that could have significant impacts on the environment and in identifying measures that departments are already putting into a plan or plan to put into place to improve their environmental performance and co-operative governance (KZN EDTEA, 2016). The agriculture and health provincial departments could use the EIP as a launching pad to develop their implementation plans detailing how policies from other sectors will impact them. From these plans, they can identify other areas of integration and collaboration. Furthermore, the agriculture, health and environment departments will need to build the capacity to co-ordinate and integrate actions, avoid replication, and implement the plans.

The provincial government’s role in food and nutrition security and the agriculture-health-environment nexus in South Africa is significant and cannot be understated. It is closer to the citizens than the national government, making it important to socioeconomic development. It is also responsible for developing and implementing a vision and framework for the province’s integrated economic, social and community development (Republic of South Africa, 1996). The provincial government is also responsible for establishing municipalities and promoting local government capacity development to enable municipalities to perform their functions and manage their affairs (Republic of South Africa, 1996). Therefore, the KZN provincial government must develop adequate policies and frameworks for food and nutrition governance that the local governments can implement under its jurisdiction, ensuring food and nutrition security at all levels and a sustainable and resilient food system.

### 3.2 SWOT analyses and way forward

SWOT analyses of the results indicate that the national and KZN provincial governments’ agriculture, health, and environment sectors have some positive attributes, including progressive policies with comprehensive aims and goals (Tables 2, 3). Some of the policies such as the *National Policy on Food and Nutrition Security (2014), Draft Climate Smart Agriculture Strategic Framework (2018), National Climate Change and Health Adaptation Plan 2014-2019, White Paper on National Climate Change Response, and the KZN Environmental Implementation Plan (EIP)*, were developed in consultation with other departments and experts, which is important for producing well-informed and inclusive policies. The willingness of experts and non-state actors to assist with policy development at national and provincial levels presents an opportunity to improve collaboration within the departments and develop strategies to implement these policies. This greatly aligns with the call for cooperative governance and intergovernmental relations within South Africa’s Constitution (Republic of South Africa, 1996). The government calls for the three spheres of government in the country to “co-operate with one another in mutual trust and good faith by (i) fostering friendly relations; (ii) assisting and supporting one another; (iii) informing one another of, and consulting one another on, matters of common interest; and (iv) co-ordinating their actions and legislation with one another” (Republic of South Africa, 1996, p. 21). The departments also acknowledge the importance of partnerships and the need to co-ordinate and harmonize policies and understand that food security is a complex problem that transcends social, health and economic boundaries and requires a comprehensive and multidisciplinary response. These are some of the foundational steps required to develop a sustainable food system while contributing to the attainment of the Sustainable Development Goals, in particular, Goals 2, 3, and 12, which relate to poverty eradication, ending hunger, achieving food security and improved nutrition, and promoting sustainable agriculture, as well as ensuring responsible consumption and production patterns.

Conversely, the sectors possess many negative attributes due to internal factors, while others are due to external factors, which are not in the control of the departments but affect them (Yukalang et al., 2017). At the national level, the main weaknesses include the lack of recognition of cross-linkages between the three sectors and the lack of collaboration and coordination between them due to ineffective communication and the fact that the departments are primarily focused on their core mandates (Momberg et al., 2020). This focus restricts their ability to address cross-cutting issues outside their immediate obligations (Momberg et al., 2020). There is also a low capacity for cross-sectoral planning and a lack of coordination between the national, provincial and local governments. Furthermore, the health sector only recently started incorporating environmental and agricultural considerations in 2010, meaning that the sector has not made much progress with holistically addressing food security.

South Africa’s government has been re-organized twice since 1994, in 2009 and recently in May 2019. This threatens how the different sectors are governed, especially the environmental and agricultural sectors. The re-organizations have likely caused tensions and frustrated some agendas and their resources. For example, the newly structured Department of Agriculture, Land Reform and Rural Development (DALRRD) may no longer have the mandate to implement its aim to develop “effective adaptation responses and increase adaptive capacity to reduce vulnerability and increase the overall resilience of South Africa’s Agriculture, Forestry and Fisheries systems, including their socio-economic and institutional characteristics” (DAFF, 2018, p. 5) as listed in its 2018 Draft Climate Smart Agriculture Strategic Framework. This, therefore, calls for greater collaboration between the DEFF and the DALRRD. The re-organizations may have also hindered the plans of the Department of Environmental Affairs and Tourism to eliminate current fragmentation, duplication and lack of co-ordination by reviewing all existing legislation and preparing a single piece of legislation dealing with all waste and pollution matters, as stated in the *White Paper on Integrated Pollution and Waste Management (2000)*. The re-organizations can also lead to policy uncertainty and administrative turmoil, undermining the successful, incremental translation of policy into action and hindering transformative change (Witt, 2018). Naidoo (2018, p. 3) concurs, stating that reorganizing government machinery can also contribute to “sharpening the divide between policy and implementation”. Other threats to the governance of the agriculture-environment-health nexus include overlapping mandates of the different departments, which leads to a lack of coordination, duplication of policy efforts, and a lack of political will. Furthermore, South African policymakers’ short political lives (5 years) make it challenging to plan for and garner support for medium-to-longer-term agendas such as planning for sustainable food systems (Lethoko, 2016).

Within KZN, most provincial policies do not explicitly mention how health, environment and agriculture are interlinked nor address inter-sectoral coordination of activities. This is a major shortcoming of the sectoral policies, and fragmented governance threatens the agriculture-environment-health nexus and provincial food security.

The national and provincial governments need to capitalize on their strengths and take advantage of their available opportunities. Actions must be taken to overcome their weaknesses, such as increasing the capacity for cross-sectoral planning and improving interdepartmental communication and coordination. All these actions need to be taken in the face of the threats to departments, and steps need to be taken to minimize these threats’ impacts.

### 3.3 Analysis of the systemic structure of the status quo in the food policy systems of the most vulnerable

**Figure 1:**
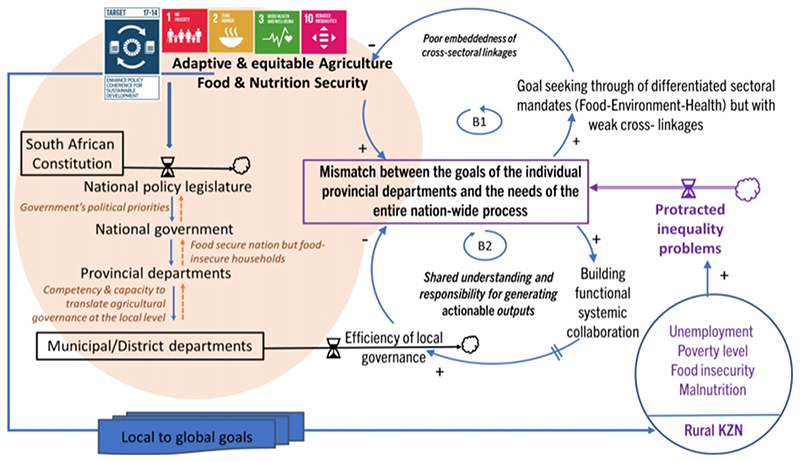
Coupled balancing feedback loops comparing pathways that deter (Bl) or enhance (B2) achievement food and nutrition security governance goals between KwaZulu Natal (KZN) provincial and national policy making. The UNSDGs elaborates the overarching local to global goals that governments ought to achieve. The South African government establishes its priorities through the policy legislature. The anomaly that occurs is that although South Africa is a food-secure country, household food security remain rampant. Target 17.14 of the United Nations Sustainable Development Goals emphasizes the need for countries to create governance mechanisms that can tackle cross-cutting issues when dealing with systemic transitions such as that required to make South Africa’s food system inclusive and secure. The main goal is to nurture an adaptive and equitable agricultural sector that guarantees food and nutrition security (FNS) through adequate interactions with other relevant sectors such as environment and health. Loop Bl represents current limitations that result in poor embeddedness of cross-sectoral in policy responses despite the existence of various policy documents in the food, environmental, and health sectors. The aim of Loop B2 is to promote Policy Coherence for Sustainable Development (PCSD) as stipulated in UN Goal 17 which should occur at all governance levels, horizontally (across sectors) and vertically (across governance levels). Promoting B2 does not mean that Bl will be tackled linearly. For this reason, policy-making at various department levels and sectors ought to regard their policy documents as living documents to ensure that their response mechanisms are adaptive and coherent in tackling the dynamics of food security issues. An “equal” sign on an arrow denotes systemic delay in the causal relationship.

Figure 1 shows that embracing collaborative processes is key so that stakeholders in policy governance can co-design and co-produce pathways to tackle intractable societal challenges. Wicked and protracted inequality problems such as unemployment, poverty, food insecurity and malnutrition increase the severity of societal challenges and inequality. The expanding inequality makes it difficult for national policies, which were made exante compared to the evolving issues, to respond, intervene, and regulate societal challenges at local levels. Consequently, there is an accumulation of goal achievement mismatch between national and provincial governments. The current state of policy integration operates in a balancing loop that limits the achievement of an adaptive and equitable national food security level because cross-linkages, while being acknowledged, are not coherently embedded in policy governance. Weak policy integration results in situations that drift away from the main goal, as shown in B1.

The balancing loop B2 is presented to counter-balance the impact of the mismatch. The intent is to create shared understanding, especially in terms of contextual interpretations of problems and responsibility for generating actionable policy outputs. Building functional and systemic collaborative mechanisms and dialogue processes such as those prescribed by the United Nations SDG Target 17.14 (OECD, 2016) could act as a strong leverage point to improve the efficiency of local governance, reduce mismatch amongst the governance levels and, therefore, bring more coherence in actions and goals. The need for coherence is not restricted to Goal 17, but it acts as a thread to benchmark whether integrated approaches are being adopted to address the deep interconnections, cross-cutting elements and context-specificity in the food-environment-health sectors, which in the current case aims to promote food security, inclusivity and justice in the dual agricultural sector of post-apartheid South Africa.

The aim of building systemic functional collaboration is to enhance analytical frameworks that will help to identify policy coherence issues, and improve understanding on the food-environment-health interactions and their implications, and how certain policy actions might support or hinder desirable policy outcomes. Alignment of the existing institutional mechanisms in the three sectors can thus be improved through dialogue process and collaborations. Further, monitoring frameworks ought to consider the synergies and trade-offs when tracking progress on coherence with a view to support the national and provincial efforts for monitoring and reporting outcomes. The aim of the present work is to demonstrate the systemic structure of policy gaps or mismatch. Further analytical work is required to identify means of implementing policy coherence and coordinating mechanisms to address food security issues at the Provincial level in KZN.

## 4 Policy implications and recommendations

The study has shown that South Africa has started the conversation around the complex interlinkages and interrelationships between agriculture, health and the environment within the context of food insecurity, both at national and provincial levels. However, while the sectoral policies mention the right words, such as “co-operative governance”, “collaboration”, “holistic”, “multisectoral”, and “multi-faceted”, the various government departments are ill-equipped to develop strategies to carry out their own, often ambitious, objectives, let alone incorporate the concerns of other policy domains into their mandates, especially in a country like South Africa which is rife with so many socio-economic injustices. The government needs to emphasize and invest more in policy integration, coordination of activities, and building capacity to transition from knowledge to effective actions regarding reducing the high levels of hunger in the country and improving food and nutrition security. Conducting socio-economic impact assessments of all policy initiatives could help make the policies more effective and fulfill this goal (DPME, 2015). While South Africa has made significant progress in hunger reduction, reducing its Global Hunger Index (GHI) score from 22.7 (severe hunger) in 2005 to 14.0 (moderate severity) in 2019 (Grebmer et al., 2019), the country’s population is growing rapidly, and this will result in an unsustainable growing demand for food and competition for resources such as land and water, which will pose an additional threat to future food and nutrition security (Godfray et al., 2010; Goldblatt, 2010; Statistics South Africa, 2018). Moreover, the COVID-19 pandemic has presented new challenges by disrupting food systems.

Policymakers must decide whether to invest resources to coordinate and integrate policies or use the resources to deliver services to the increasing population (Candel, 2019). However, investing in policy integration should not be at the cost of service delivery. A cost-benefit analysis needs to be undertaken to determine at what scale the benefits of integration will outweigh the costs. It may also be beneficial to incentivize policy integration and coordination, given its potential to maximize public health and environmental outcomes. It is also necessary to address food and nutrition insecurity drivers, including poverty, inequality, and unemployment.

The agriculture, health, and environment sector actors need to develop a community of practice to address the systemic complexity of food and nutrition security to integrate and implement their policies. This constant interaction and consultation between the actors may improve coherence while minimizing contradictions and trade-offs (FAO, 2011) and promoting accountability. Furthermore, implementing policy actions needs to be followed by systematic surveillance and evaluation to review progress and guide further efforts (Mozaffarian et al., 2018). To ensure policy implementation, the national government must also hold the provincial government accountable for food and nutrition governance.

The three sectors also need to form community partnerships. This has proved successful for the KZN provincial government, which launched the flagship programme, Operation Sukuma Sakhe, in 2009 (UNAIDS Country Office South Africa and KZN Office of the Premier undated). The initiative, which means “Stand up and build”, was launched to address many social ills plaguing the province (UNAIDS Country Office South Africa and KZN Office of the Premier undated). These include, *inter alia*, poverty, food security, disease and infection (namely, HIV and TB), disempowerment of women and youth, poverty, violence against women and girls, teenage pregnancy, substance abuse, crime, and motor vehicle accidents (UNAIDS Country Office South Africa and KZN Office of the Premier undated). The project’s premise is community partnerships whereby the government works with the people to tackle the challenges they are facing (UNAIDS Country Office South Africa and KZN Office of the Premier undated). The project provides a war room, a space for the government to provide the communities with information about the services they can expect to receive. The community, in turn, provides the government with feedback on the services they are receiving and their needs (UNAIDS Country Office South Africa and KZN Office of the Premier undated). The agriculture, environment and health sectors could benefit from such a holistic approach as this will greatly enrich their policies and help them to implement the policies in a manner that is beneficial for all.

The importance of comprehensive national- and provinciallevel food system governance frameworks cannot be overstated. These frameworks set the pace for local-level governance in the country. In the absence of a champion or political will and capacity to spearhead responses at the local level, local governments need to be able to depend on these frameworks to guide them.

## 5 Conclusion

This paper explored the interlinkages that exist between the siloed sectors of agriculture, environment and health in South Africa in the context of food and nutrition security and the extent to which these interlinkages have been integrated into the country’s sector-based policies, plans, strategies and programmes, at national and provincial levels. At the provincial level, the focus of the study was on the KwaZulu-Natal province, which has the second-highest population of the nine provinces in the country. It was found that at the national level, the different sectors are aware of the interlinkages and have mentioned them on paper; however, only 17 of the 210 policy documents assessed have discussed the interlinkages in the context of food and nutrition security. Within KZN, only one of the nine policies assessed has made an effort to integrate other sectors in domains where the sectors influence each other such as food and nutrition security. Given the country’s high levels of household hunger, the rapid increase of diet-related non-communicable diseases, and the COVID-19 pandemic, wicked problems such as food and nutrition security need to be incorporated into more policies and addressed. Ending global hunger by 2030 starts with the right policies and multifaceted, coordinated and inclusive governance. Governance can drive or stall a process; therefore, good policies and political will are crucial. Transitioning to sustainable and healthy food systems will only be possible after gaps in policy and implementation have been closed, and implementation optimization has been achieved.

It is recommended that governments at all levels—national, provincial, and local need to place greater emphasis on and invest more in policy integration, co-ordination of activities, and building capacity to transition from knowledge to effective actions; decide whether to invest resources to co-ordinate and integrate policies or use the resources to deliver the services to the increasing population; develop a community of practice to address the systemic complexity of food and nutrition security to integrate and implement their policies; and conduct a socio-economic impact assessment of the policies to ascertain their effectiveness.

The extent to which the policies have been implemented is unknown, as this was beyond the scope of the paper. However, future research can be conducted on a sector-by-sector basis to investigate.

## Supplementary Material

Supplementary materials Table 1

## Figures and Tables

**Table 1:** Policies that incorporate Agriculture (including land and water), Health, and Environment.

Policy	Vision/aim/goals objectives of policy	Secto r/gove rning department
National Climate Change and Health Adaptation Plan (2014-2019)	To provide a broad framework for health sector action toward implementation of the National Climate Change Response Policy (NCCRP) To effectively manage inevitable climate change impacts on health through interventions that build and sustain South Africa’s socio-economic and environmental resilience and emergency response capacity Describe the environmental and health contexts for the proposals contained in this plan Outline a broad programme of activities to be undertaken or spearheaded by the South African health sector, giving specific examples Indicate the potential partners, time frames and financial implications	Health
White Paper on Agriculture (1995)	To ensure equitable access to agriculture and promote the contribution of agriculture to the development of all communities, society at large and the national economy, in order to enhance income, food security, employment and quality of life in a sustainable manner.	Agriculture
Water Services Act (No. 108 of 1997)	To provide for the rights of access to basic water supply and basic sanitation To provide for the setting of national standards and of norms and standards for tariffs To provide for water services development plans; to provide a regulatory framework for water services institutions and water services intermediaries To provide for the establishment and disestablishment of water boards and water services committees and their powers and duties; to provide for the monitoring of water services and intervention by the Minister or by the relevant Province To provide for financial assistance to water services institutions To provide for certain general powers of the Minister To provide for the gathering of information in a national information system and the distribution of that information To repeal certain laws; and To provide for matters connected therewith	Agriculture
Pesticide Management Policy for South Africa (2010)	To improve legislative framework to ensure that South Africans are better protected from health and environmental risks posed by pesticides To encourage the development and use of alternative products and techniques and reduce dependence on chemical plant protection products To integrate relevant international agreements and initiatives from other government departments Increased transparency. access to information and improve public participation in the registration of pesticides	Agriculture
Integrated growth and development plan (IGDP) for agriculture, forestry and fisheries (2012)	To promote equitable, productive, competitive, profitable and sustainable agriculture, forestry and fisheries sectors, growing to the benefit of all South Africans	Agriculture
Agricultural Policy Action Plan (APAP) (2014-2019)	The APAP seeks to translate the high-level responses offered in the Integrated growth and development plan (IGDP) for agriculture, forestry and fisheries (2012) into tangible, concrete steps The APAP is planned over a five-year period and will be updated on an annual basis Aligning itself with the New Growth Path (NGP), the National Development Plan (NDP) and Industrial Policy Action Plan (IPAP), APAP seeks to assist in the achievement of Outcome 4, Decent Employment through Inclusive Growth, and that of Outcome 7, Comprehensive Rural Development and Food Security	
National Policy on Food and Nutrition Security (2014)	To ensure the accessibility and affordability of safe and nutritious food at national and availability, household levels	Agriculture
Draft Climate Smart Agriculture Strategic Framework (2018)	To promote effective adaptation responses and increase adaptive capacity in order to reduce vulnerability and increase overall resilience of South Africa’s Agriculture, Forestry and Fisheries (AFF) systems, including their socio-economic and institutional characteristics	Agriculture
Draft White Paper on Conservation and Sustainable Use of Biodiversity (1997)	VISION: A prosperous, environmentally conscious nation, whose people are in harmonious co-existence with the natural environment, and which derives lasting benefits from the conservation and sustainable use of its rich biological diversity. In addition, the Paper states that because of the cross-sectoral nature of biodiversity, several other national government departments will play a vital role in the implementation of this policy. These include the Departments of Agriculture; Land Affairs; Water Affairs and Forestry; Trade and Industry; Foreign Affairs; Health; Transport; Housing; Welfare and Population Development; Arts, Culture, Science and Technology; Finance; as well as the South African National Defence Force. Of crucial importance will be their commitment to cooperating with one another, and to developing sectoral-specific plans and budgets to reflect how biodiversity considerations will be incorporated into the activities of departments.	Environment
White Paper on Environmental Management Policy (1998)	VISION: to unite the people of South Africa in working toward a society where all people have sufficient food, clean air and water, decent homes and green spaces in their neighborhoods enabling them to live in spiritual, cultural and physical harmony with their natural surroundings	Environment
White Paper on Integrated Pollution and Waste Management (2000)	VISION: To develop, implement and maintain an integrated pollution and waste management system which contributes to sustainable development and a measurable improvement in the quality of life, by harnessing the energy and commitment of all South Africans for the effective prevention, minimization and control of pollution and water.	Environment
National Biodiversity Strategy and Action Plan (2005)	Conserve and manage terrestrial and aquatic biodiversity to ensure sustainable and equitable benefits to the people of South Africa, now and in the future	Environment
National Framework for Sustainable Development (July 2008)	VISION: South Africa aspires to be a sustainable, economically prosperous and self-reliant nation state that safeguards its democracyby meeting the fundamental human needs of its people, by managing its limited ecological resources responsibly for current and future generations, and by advancing efficient and effective integrated planning and governance through national, regional and global collaboration.	Environment
White Paper on National Climate Change Response (NCCR) (2011)	To effectively manage inevitable climate change impacts through interventions that build and sustain South Africa’s social, economic and environmental resilience and emergency response capacity To make a fair contribution to the global effort to stabilize GHG concentrations in the atmosphere at a level that avoids dangerous anthropogenic interference with the climate system within a timeframe that enables economic, social and environmental development to proceed in a sustainable manner	Environment
National Strategy for Sustainable Development and Action Plan (2011–2014) (NSSD 1)	South Africa aspires to be a sustainable, economically prosperous and self-reliant nation that safeguards its democracy by meeting the fundamental human needs of its people, by managing its limited ecological resources responsibly for current and future generations, and by advancing efficient and effective integrated planning and governance through national, regional and global collaboration	Environment
South Africa’s National Biodiversity Framework (2019–2024)	Conserve, manage, and sustainably use biodiversity to ensure benefits to the people of South Africa, now and in the future	Environment
**KZN**		
KZN Environmental Implementation Plan (EIP)	To co-ordinate and harmonize the environmental policies, plans, programmes and decisions of the various national departments that exercise functions that mayaffect the environment or are entrusted with powers and duties aimed at the achievement, promotion, and protection of a sustainable environment, and of provincial and local spheres of government, in order to: (i) Minimize the duplication of procedures and functions; and (ii) Promote consistency in the exercise of functions that may affect the environment; Give effect to the principle of co-operative government in chapter 3 of the Constitution Secure the protection of the environment across the country as a whole Prevent unreasonable actions by provinces in respect of the environment that are prejudicial to the economic or health interests of other provinces or the country as a whole; and Enable the Minister to monitor the achievement, promotion, and protection of a sustainable environment	KZN Environment

**Table 2:** SWOT analysis for national government.

	Positive	Negative
**Internal**	**STRENGTHS** Progressive policies with comprehensive aims and goals Consultation of a multi-disciplinary team of experts in developing policies Recognition of the need for inter- and intra-departmental collaboration Acknowledgment of the challenges that exist in achieving departmental goals Recognition of the need for cooperation and inter-sectoral collaborations, as well as for partnerships and dialogue between state and non-state actors	**WEAKNESSES** Lack of recognition of crosslinkages that exist between the health, environment and agriculture sectors Lack of co-ordination between national, provincial and local government Limited inter-departmental and intradepartmental co-ordination Low capacity for cross-sectoral planning Ineffective communication between sector departments Collaboration and cooperation efforts on policies, programmes and plans are weak Delayed action- the health sector only recently started incorporating environmental and agricultural considerations in 2010
**External**	**OPPORTUNITIES** Willingness of experts to assist with policy development	**THREATS** Reconfiguration of departments by the President Fragmented governance Overlapping mandates of different government institutions Lack of political commitment/leadership/will Profusion of laws Duplication of efforts Lack of co-ordination and integration Five-year political term

**Table 3:** SWOT analysis for KZN provincial government.

	Positive	Negative
**Internal**	**STRENGTHS** Progressive policies with comprehensive aims and goals Reference to other sectors within policies Recognition of the need for inter- and intra-departmental collaboration Acknowledgment of the need to co-ordinate and harmonize policies Understanding that food security is a complex problem that transcends social, health and economic boundaries and requires a comprehensive and multi-disciplinary response Identification of policies, plans and programmes within each of the provincial and relevant national departments and local government that could have significant impacts on the environment by the Environmental Implementation Plan	**WEAKNESSES** No explicit mention of how the health, environment and agriculture, are interlinked Very little reference is made about inter-sectoral co-ordination of activities
**External**	**OPPORTUNITIES** Willingness of experts to assist with policydevelopment	**THREATS** Fragmented governance

## Footnotes

1 Chapter 3, Section 11(1) of the National Environmental Management Act, 1998 (Act No. 107 of 1998) (NEMA) requires that national and provincial departments exercising functions which may affect the environment, in terms of *Schedule 1* of the Act, prepare an Environmental Implementation Plan (EIP) within one year of the promulgation of this Act and at least every 5 years thereafter.
