# Supplementary material for: Policy gaps and food systems optimization: a review of agriculture, environment, and health policies in South Africa
Source: Front Sustain Food Syst. Author manuscript; Available in PMC 2023 Sep 8. (PMC7615054; doi:10.3389/fsufs.2023.867481)
Supplement: Supplementary materials Table 1 [file EMS187385-supplement-Supplementary_materials_Table_1.DOCX]

Supplementary Material

# Supplementary Information 1

**NATIONAL POLICIES ON AGRICULTURE**

Table 1 presents the policies, acts and strategies developed and published by the various departments in order to govern South Africa’s agricultural sector, directly and indirectly. The documents were reviewed to find out whether, and to what extent human health and environmental aspects were incorporated. Given the importance of water and land to agriculture, water and land are included as criteria in the table.

*Table* *1: Review of South African national agriculture-related policies and strategies*

| **Name of policy/Act/Strategy** | **Human Health/Wellbeing** | **Land** | **Water** | **Environment/Climate change/Sustainable development** |
| --- | --- | --- | --- | --- |
| Fertilizers, Farm Feeds, Agricultural Remedies and Stock Remedies Act, 1947 (Act No. 36 of 1947) (see South African animal feed policy below | 🗶 | 🗶 | 🗶 | ✓ |
| Animals Protection Act, 1962 (Act No. 71 of 1962) | 🗶 | 🗶 | 🗶 | 🗶 |
| The Agricultural Credit Act, 1966 (Act 28 of 1966) | 🗶 | ✓ | ✓ | 🗶 |
| The Subdivision of Agricultural Land Act, 1970 (Act 70 of 1970) | 🗶 | ✓ | 🗶 | 🗶 |
| Plant Breeders’ Rights Act, 1976 (Act No. 15 of 1976) | 🗶 | 🗶 | 🗶 | 🗶 |
| Plant Improvement Act, 1976 (Act No. 53 of 1976) | 🗶 | 🗶 | 🗶 | 🗶 |
| Livestock Improvement Act, 1977 (Act No. 25 of 1977) To be repealed by the Animal Improvement Act, 1998 (Act No. 62 of 1998) | 🗶 | 🗶 | 🗶 | 🗶 |
| Perishable Products Export Control Act, 1983 (Act No. 9 of 1983) | 🗶 | 🗶 | 🗶 | 🗶 |
| Agricultural Pests Act, 1983 (Act No. 36 of 1983) | 🗶 | 🗶 | 🗶 | 🗶 |
| Conservation of Agricultural Resources Act (CARA), 1983 (Act No 43 of 1983) | 🗶 | ✓ | ✓ | ✓ |
| Animal Diseases Act, 1984 (Act No. 35 of 1984) To be repealed by the Animal Health Act, 2002 (Act No. 7 of 2002) | 🗶 | 🗶 | 🗶 | 🗶 |
| The Sea Fishery Act, 1988 (Act 12 of 1988) | 🗶 | ✓ | ✓ | 🗶 |
| The Liquor Products Act, 1989 (Act 60 of 1989) | 🗶 | 🗶 | ✓ | 🗶 |
| Agricultural Research Act, 1990 (Act No. 86 of 1990) | 🗶 | 🗶 | 🗶 | ✓ |
| Agricultural Product Standards Act, 1990 (Act No. 119 of 1990) | 🗶 | 🗶 | 🗶 | 🗶 |
| Agricultural Produce Agents Act, 1992 (Act No. 12 of 1992) | 🗶 | 🗶 | 🗶 | 🗶 |
| White Paper on Agriculture (1995) | ✓ | ✓ | ✓ | ✓ |
| Genetically Modified Organisms Act, 1997 (Act No. 15 of 1997) | 🗶 | 🗶 | 🗶 | ✓ |
| Water Services Act [No. 108 of 1997] | ✓ | ✓ | ✓ | ✓ |
| The Marine Living Resources Act, 1998 (Act 18 of 1998) | 🗶 | ✓ | ✓ | ✓ |
| Animal Improvement Act, 1998 (Act No. 62 of 1998) | 🗶 | 🗶 | 🗶 | 🗶 |
| Agricultural Laws Rationalisation Act, 1998 (Act No. 72 of 1998) | 🗶 | 🗶 | 🗶 | 🗶 |
| The National Forests Act, 1998 (Act 84 of 1998) | 🗶 | ✓ | ✓ | 🗶 |
| The National Veld and Forest Fire Act, 1998 (Act 101 of 1998) | 🗶 | ✓ | 🗶 | ✓ |
| The Onderstepoort Biological Products Incorporation Act, 1999 (Act 19 of 1999) | 🗶 | 🗶 | 🗶 | 🗶 |
| Meat Safety Act, 2000 (Act No. 40 of 2000) | 🗶 | 🗶 | 🗶 | 🗶 |
| The Land Redistribution Policy for Agricultural Development (LRAD) (2001)/sub programme | 🗶 | ✓ | 🗶 | ✓ |
| Land and Agricultural Development Bank Act, 2002 (Act No. 15 of 2002) | 🗶 | ✓ | 🗶 | ✓ |
| Animal Identification Act (No.6 of 2002) | 🗶 | 🗶 | 🗶 | 🗶 |
| Disaster Management Act (Act 57 of 2002) | 🗶 | 🗶 | 🗶 | 🗶 |
| Pesticide Management Policy for South Africa (2010) | ✓ | ✓ | ✓ | ✓ |
| Integrated growth and development plan (IGDP) for agriculture, forestry and fisheries (2012) | ✓ | ✓ | ✓ | ✓ |
| The Marine Living Resources Amendment Act, 2014 (Act 5 of 2014) | 🗶 | 🗶 | ✓ | 🗶 |
| Agricultural Policy Action Plan (APAP) (2015-2019) (November 2014) | ✓ | ✓ | ✓ | ✓ |
| *National Policy on Food and*  Nutrition Security, 2014 (Developed by DAFF and Department of Social Development) | ✓ | ✓ | ✓ | ✓ |
| Preservation and Development of Agricultural Land Bill (2016) | 🗶 | 🗶 | 🗶 | ✓ |
| South African animal feed policy/ Fertilizers, Farm Feeds, Agricultural Remedies and Stock Remedies Act, 1947 | 🗶 | 🗶 | 🗶 | 🗶 |
| Draft Conservation Agriculture Policy | 🗶 | ✓ | ✓ | ✓ |
| Draft policy document on the Preservation and Development of Agricultural Land (2018) | 🗶 | ✓ | ✓ | ✓ |
| National Policy on Comprehensive Producer Development Support: Draft 5 ver. 3 (May 2018) | 🗶 | ✓ | ✓ | ✓ |
| Draft Climate Smart Agriculture Strategic Framework (August 2018) | ✓ | ✓ | ✓ | ✓ |

# Supplementary Information 2

**NATIONAL POLICIES ON HEALTH**

Table 2 presents the policies, acts and strategies developed and published by DoH to govern South Africa’s health sector, directly and indirectly.

**Table** **2:** Review of South African national health-related policies and strategies

| **Name of policy** | **Agriculture/Food** | **Land** | **Water** | **Environment/Climate change/Sustainable development** |  |
| --- | --- | --- | --- | --- | --- |
| The Medicines and Related Substances Act, 1965 (Act 101 of 1965) | ✓ | 🗶 | 🗶 | 🗶 |  |
| The Foodstuffs, Cosmetics and Disinfectants Act, 1972 (Act 54 of 1972 | ✓ | 🗶 | 🗶 | 🗶 |  |
| Hazardous Substances Act, 1973 (Act 15 of 1973) | 🗶 | 🗶 | 🗶 | 🗶 |  |
| Occupational Diseases in Mines and Works Act, 78 of 1973 | 🗶 | 🗶 | 🗶 | 🗶 |  |
| The Pharmacy Act, 1974 (Act 53 of 1974) | 🗶 | 🗶 | 🗶 | 🗶 |  |
| Health Professions Act, 56 of 1974 (as amended) | 🗶 | 🗶 | 🗶 | 🗶 |  |
| The Criminal Procedure Act, 1977 (Act 51 of 1977), Sections 212 4(a) and 212 8(a) | 🗶 | ✓ | 🗶 | 🗶 |  |
| The Dental Technicians Act, 1979 (Act 19 of 1979) | 🗶 | 🗶 | 🗶 | 🗶 |  |
| Allied Health Professions Act, (Act 63 of 1982 (as amended) | 🗶 | 🗶 | 🗶 | 🗶 |  |
| Human Tissue Act, 65 of 1983 | 🗶 | 🗶 | 🗶 | 🗶 |  |
| National Policy for Health Act, 116 of 1990 | 🗶 | 🗶 | 🗶 | 🗶 |  |
| South African Medical Research Council (SAMRC) Act, 58 of 1991 | 🗶 | 🗶 | 🗶 | 🗶 |  |
| Academic Health Centres Act, 86 of 1993 | 🗶 | 🗶 | 🗶 | 🗶 |  |
| The Tobacco Products Control Act, 1993 (Act 83 of 1993) | 🗶 | 🗶 | 🗶 | 🗶 |  |
| The Occupational Health and Safety Act, 1993 (Act 85 of 1993) | 🗶 | 🗶 | 🗶 | 🗶 |  |
| Radiation Control - Act: Group IV Hazardous Substances - Exclusions and Exemptions (1993) | 🗶 | 🗶 | 🗶 | 🗶 |  |
| The Compensation for Occupational Injuries and Diseases Act, 1993 (Act 130 of 1993) | 🗶 | 🗶 | 🗶 | 🗶 |  |
| The Choice on Termination of Pregnancy Act, 92 of 1996 (as amended) | 🗶 | 🗶 | 🗶 | 🗶 |  |
| The National Roads Traffic Act, 1996 (Act 93 of 1996) | 🗶 | 🗶 | 🗶 | 🗶 |  |
| Genetically Modified Organisms Act (No. 15 of 1997) | 🗶 | 🗶 | 🗶 | 🗶 |  |
| Nursing Amendment Act (Act 19 of 1997) | 🗶 | 🗶 | 🗶 | 🗶 |  |
| Compensation for Occupational Injuries and Diseases Amendment Act (Act 61 of 1997) | 🗶 | 🗶 | 🗶 | 🗶 |  |
| Dental Technicians Amendment Act (Act 43 of 1997) | 🗶 | 🗶 | 🗶 | 🗶 |  |
| The Labour Relations Act, 1995 (Act 66 of 1995) | 🗶 | 🗶 | 🗶 | 🗶 |  |
| The National Roads Traffic Act, 1996 (Act 93 of 1996) | 🗶 | 🗶 | 🗶 | 🗶 |  |
| White Paper for the transformation of the health system in South Africa (1997) | ✓ | 🗶 | ✓ | ✓ |  |
| Pharmacy Amendment Act (Act 88 of 1997) | 🗶 | 🗶 | 🗶 | 🗶 |  |
| Medical, Dental, and Supplementary Health Service Professions Amendment Act (Act 89 of 1997) | 🗶 | 🗶 | 🗶 | 🗶 |  |
| Medicines and Related Substances Control Amendment Act (Act 90 of 1997) | 🗶 | 🗶 | 🗶 | 🗶 |  |
| Chiropractors,Homeopaths Substances and Allied Health Service Professions Amendment Act (Act 91 of 1997) | 🗶 | 🗶 | 🗶 | 🗶 |  |
| The Medical Schemes Act, 1998 (131 of 1998) | 🗶 | 🗶 | 🗶 | 🗶 |  |
| The Skills Development Act, 1998 (Act 97of 1998) | 🗶 | 🗶 | 🗶 | 🗶 |  |
| The Sterilisation Act (Act 44 of 1998) | 🗶 | 🗶 | 🗶 | 🗶 |  |
| The Employment Equity Act, 1998 (Act 55 of 1998) | 🗶 | 🗶 | 🗶 | 🗶 |  |
| The State Information Technology Act, 1998 (Act 88 of 1998) | 🗶 | 🗶 | 🗶 | 🗶 |  |
| The Public Finance Management Act, 1999 (Act 1 of 1999) | 🗶 | 🗶 | 🗶 | 🗶 |  |
| The Tobacco Products Control Amendment Act, 12 of 1999 (as amended) | 🗶 | 🗶 | 🗶 | 🗶 |  |
| The Promotion of Access to Information Act, 2000 (Act 2 of 2000) | 🗶 | 🗶 | 🗶 | ✓ |  |
| The Promotion of Administrative Justice Act, 2000 (Act 3 of 2000) | 🗶 | 🗶 | 🗶 | 🗶 |  |
| The Promotion of Equality and the Prevention of Unfair Discrimination Act, 2000 (Act 4 of 2000) | 🗶 | ✓ | 🗶 | 🗶 |  |
| National Health Laboratory Service (NHLS), 2000 (Act 37 of 2000) | 🗶 | 🗶 | 🗶 | 🗶 |  |
| The Council for Medical Schemes (CMS) Levy Act, 2000 (Act 58 of 2000) | 🗶 | 🗶 | 🗶 | 🗶 |  |
| Pharmacy Amendment Act (Act 1 of 2000) | 🗶 | 🗶 | 🗶 | 🗶 |  |
| The Mental Health Care Act, 2002 (Act 17 of 2002) | 🗶 | 🗶 | 🗶 | 🗶 |  |
| The Broad-Based Black Economic Empowerment Act, 2003 (Act 53 of 2003) | 🗶 | 🗶 | 🗶 | ✓ |  |
| Medicines and Related Substances Amendment Act 59 of 2002 | 🗶 | 🗶 | 🗶 | 🗶 |  |
| National Health Act (no. 61 of 2003) | 🗶 | 🗶 | 🗶 | ✓ |  |
| The Nursing Act, 2005 (Act 33 of 2005) | 🗶 | 🗶 | 🗶 | 🗶 |  |
| The Children’s Act, 2005 (Act 38 of 2005) | 🗶 | 🗶 | 🗶 | 🗶 |  |
| The Traditional Health Practitioners Act, 2007 (Act 22 of 2007) | 🗶 | 🗶 | 🗶 | 🗶 |  |
| National Department of Health Strategic Plan 2010/11-2012/13 | ✓ | 🗶 | 🗶 | ✓ |  |
| National Mental Health Policy Framework and Strategic Plan 2013-2020 | 🗶 | ✓ | 🗶 | 🗶 |  |
| National Environmental Health Policy, December 2013 | ✓ | ✓ | ✓ | ✓ |  |
| Roadmap for Nutrition in South Africa  2013 - 2017 | ✓ | 🗶 | ✓ | 🗶 |  |
| Strategic Plan for the Prevention and Control of Non-Communicable Diseases 2013-17 | ✓ | 🗶 | 🗶 | ✓ |  |
| National Department of Health  Strategic Plan 2014/15 to 2018/19 | ✓ | 🗶 | 🗶 | ✓ |  |
| National Climate Change and Health Adaptation Plan 2014-2019 | ✓ | ✓ | ✓ | ✓ | ✓ |
| Republic of South Africa Department of Health Strategic Plan 2015/16 - 2019/20 | ✓ | 🗶 | ✓ | ✓ |  |
| National Guideline for Patient Safety Incident Reporting and Learning in the Public Health Sector of South Africa, March 2017 | 🗶 | 🗶 | 🗶 | ✓ |  |
| National Public Health Institute of South Africa Bill (2017) | 🗶 | 🗶 | 🗶 | 🗶 |  |

# Supplementary Information 3

**NATIONAL POLICIES ON ENVIRONMENT**

**Table 3:** Review of South African national environment-related policies and strategies

| **Name of policy** | **Agriculture/Food** | **Human Health/Wellbeing** | **Land** | **Water** |
| --- | --- | --- | --- | --- |
| The Prince Edward Islands Act, 1948 (Act 48 of 1948) | 🗶 | 🗶 | 🗶 | 🗶 |
| Atmospheric Pollution Prevention Act (No. 45 of 1965) | 🗶 | ✓ | ✓ | 🗶 |
| The Sea Birds and Seals Protection Act 1973 (Act 46 of 1973) | 🗶 | 🗶 | 🗶 | ✓ |
| The Sea Fishery Act, 1988 (Act 12 of 1988) | ✓ | 🗶 | 🗶 | ✓ |
| Environment Conservation Act (No. 73 of 1989) | 🗶 | 🗶 | 🗶 | 🗶 |
| Sea Fishery Amendment Act, 1992 (Act No. 57 of1992) | 🗶 | 🗶 | 🗶 | ✓ |
| Environment Conservation Amendment Act, (No. 79 of 1992) | 🗶 | 🗶 | 🗶 | 🗶 |
| Sea-Shore Amendment Act (No. 190 of 1993) | 🗶 | 🗶 | 🗶 | 🗶 |
| Restitution of Land Rights Act, 1994 (Act No. 22 of 1994) | ✓ | 🗶 | ✓ | 🗶 |
| Dumping at Sea Control Amendment Act, (No. 73 of 1995) | 🗶 | 🗶 | 🗶 | ✓ |
| Trust Property Control Act, 1988 (Act No. 57 of 1988) as amended by Justice Law Realisation Act, 1996 (Act No. 18 of 1996) | 🗶 | 🗶 | 🗶 | 🗶 |
| The Antarctic Treaties Act, (Act No. 60 of 1996) November 1996 | 🗶 | 🗶 | 🗶 | 🗶 |
| Environmental Laws Rationalisation Act, (No. 51 of 1997) | 🗶 | 🗶 | 🗶 | 🗶 |
| National Parks Amendment Act, (No. 70 of 1997) | 🗶 | 🗶 | 🗶 | 🗶 |
| The White Paper on Conservation and Sustainable Use of Biodiversity, 1997 | ✓ | ✓ | ✓ | ✓ |
| Marine Living Resources Act, (No. 18 of 1998) | 🗶 | 🗶 | ✓ | ✓ |
| National Water Act, 1998 (No. 36 of 1998) | 🗶 | ✓ | ✓ | ✓ |
| National Parks Amendment Act (No. 106 of 1998) | 🗶 | 🗶 | ✓ | 🗶 |
| The White Paper on Environmental Management, 1998 | ✓ | ✓ | ✓ | ✓ |
| National Environmental Management Act (No. 107 of 1998) (NEMA) | 🗶 | ✓ | ✓ | ✓ |
| Public Finance Management Act (No. 1 of 1999) | 🗶 | 🗶 | 🗶 | 🗶 |
| World Heritage Convention Act (No. 49 of 1999) | ✓ | 🗶 | ✓ | ✓ |
| Promotion of Administrative Justice Act 2000 (Act No. 3 of 2000) | 🗶 | 🗶 | 🗶 | 🗶 |
| The White Paper on Integrated Pollution and Waste Management, 2000 | ✓ | ✓ | ✓ | ✓ |
| The White Paper for Sustainable Coastal Development in South Africa, 2000 | ✓ 🗶 | ✓ 🗶 | ✓ | ✓ |
| South African Weather Service (SAWS) Act, 2001 (Act 8 of 2001) | ✓ | 🗶 | 🗶 | ✓ |
| National Parks Amendment Act (December 2001) | 🗶 | 🗶 | 🗶 | 🗶 |
| National Environmental Management Act: Regulations: Control of vehicles in coastal zone (December 2001) | 🗶 | ✓ | ✓ | ✓ |
| Promotion of Access to Information Act (Act No. 2 of 2002) | 🗶 | ✓ | 🗶 | 🗶 |
| Environment Conservation Act: Regulations: Activities identified under section 21 (May 2002) | 🗶 | 🗶 | 🗶 | 🗶 |
| National Environmental Management Act Amendment (No. 56 of 2002) | 🗶 | 🗶 | 🗶 | 🗶 |
| Broad-Based Black Economic Empowerment Act (No. 53 of 2003) (January 2004) | 🗶 | 🗶 | 🗶 | 🗶 |
| National Environmental Management Act Amendment (No. 46 of 2003) (February 2004) | 🗶 | 🗶 | ✓ | 🗶 |
| National Environmental Management Act: Protected Areas Act (No. 57 of 2003) (February 2004) | 🗶 | 🗶 | ✓ | ✓ |
| Environment Conservation Amendment Act (No. 50 of 2003) (February 2004) | 🗶 | ✓ | 🗶 | ✓ |
| NEM: Biodiversity Act (No. 10 of 2004) | ✓ | ✓ | ✓ | 🗶 |
| National Environmental Management Act Amendment (No. 8 of 2004) | 🗶 | 🗶 | 🗶 | 🗶 |
| National Environmental Management Act, 1998 (Act No. 107 of 1998): Amendments to Regulation published in terms of Section 44 of NEMA: Control of Use of Vehicles in the Coastal Zone (GN Regulation 1399 of 21 December 2001) (December 2004) | 🗶 | 🗶 | ✓ | ✓ |
| National Environmental Management Act: Regulations: Control of use of vehicles in coastal zone: Guidelines on implementation of regulations (December 2004) | 🗶 | ✓ | 🗶 | 🗶 |
| National Environmental Management Act: Regulations: Establishment of a designated national authority for the clean development mechanism (December 2004) | 🗶 | 🗶 | 🗶 | 🗶 |
| National Environmental Management: Biodiversity Act: Regulations: Keeping and hunting of acinonyx jubatus, hyaena brunnea, crocuta, lycaon pictus, panthera leo and panthera pardus (January 2005) | 🗶 | 🗶 | 🗶 | 🗶 |
| National Environmental Management: Protected Areas Amendment Act, (No. 31 of 2004), G 27274 (02 February 2005) | 🗶 | 🗶 | 🗶 | ✓ |
| NEM: Air Quality Act (No. 39 of 2004) (February 2005) | 🗶 | ✓ | 🗶 | 🗶 |
| National Biodiversity Strategy and Action Plan (2005) | ✓ | ✓ | ✓ | ✓ |
| Environment Conservation Act: Regulation: Waste tyre (February 2007) | 🗶 | 🗶 | ✓ | ✓ |
| National Environmental Management: Biodiversity Act: Amendment to Threatened or protected species regulations (December 2007) | 🗶 | 🗶 | 🗶 | 🗶 |
| Maritime Living Resources Act: Regulations (January 2008) | 🗶 | 🗶 | 🗶 | 🗶 |
| National Environmental Management: Biodiversity Act: Regulations: Threatened or protected species: Amendment (January 2008) | 🗶 | 🗶 | ✓ | 🗶 |
| February 2008 National Environmental Management: Biodiversity Act (10/2004): Bio-Prospecting, Access and Benefit-Sharing Regulations (February 2008) | 🗶 | 🗶 | 🗶 | 🗶 |
| National Environmental Management: Biodiversity Act: Regulations: Bio-prospecting, access and benefit-sharing (February 2008) | 🗶 | 🗶 | 🗶 | 🗶 |
| Marine Living Resources Act: Regulations: Amendment (March 2008) | 🗶 | 🗶 | 🗶 | 🗶 |
| National Environmental Management: Biodiversity Act: Regulations: Threatened or protected species (January 2008) | 🗶 | 🗶 | ✓ | 🗶 |
| Environment Conservation Act 1989: Regulationsfor Prohibition of use, manufacturing, import and export of asbestos and asbestos containing materials (March 2008) | 🗶 | 🗶 | 🗶 | 🗶 |
| Marine Living Resources Act: Regulations: Management of boat based whale watching and protection of turtles (July 2008) | 🗶 | 🗶 | 🗶 | 🗶 |
| Marine Living Resources Act: Regulations: Management of white shark cage diving (July 2008) | 🗶 | 🗶 | 🗶 | 🗶 |
| National Framework for Sustainable Development (July 2008) | ✓ | ✓ | ✓ | ✓ |
| National Environmental Management: Biodiversity Act: Regulations: Threatened or protected species: 2nd amendment (August 2008) | 🗶 | 🗶 | 🗶 | 🗶 |
| Marine Living Resources Act: Regulations: Stilbaai Marine Protected Area (October 2008) | 🗶 | 🗶 | 🗶 | ✓ |
| National Environmental Management Act: Environmental Laws Amendment (December 2008 Act 44 of 2008) | 🗶 | 🗶 | 🗶 | 🗶 |
| No.62 of 2008: National Environmental Management Amendment Act, 2008 (No. 31789)(January 2009) | 🗶 | 🗶 | ✓ | ✓ |
| NEM: Integrated Coastal Management Act (No. 24 of 2008) (February 2009) | 🗶 | ✓ | ✓ | ✓ |
| Environment Conservation Act: Regulations: Waste tyre (February 2009) | ✓ | ✓ | 🗶 | ✓ |
| National Environmental Management: Biodiversity Act: Regulations: Threatened or protected species: 2nd amendment (February 2009) | 🗶 | 🗶 | ✓ | 🗶 |
| National Environmental Management: Biodiversity Act: Regulations: Threatened or protected species: Amendment (February 2009) | 🗶 | 🗶 | 🗶 | 🗶 |
| Marine Living Resources Act: Regulations: Amendment (March 2009) | 🗶 | 🗶 | 🗶 | 🗶 |
| National Environmental Management of Waste Act (Act 59 of 2008) (March 2009) | 🗶 | ✓ | ✓ | ✓ |
| Mineral and Petroleum Resources Development Act, 2008 (Act No.49 of 2008) | 🗶 | ✓ | ✓ | ✓ |
| National Environmental Management Act: Environmental Laws Amendment, (No 14 of 2009) (May 2009) | ✓ | ✓ | ✓ | 🗶 |
| National Environmental Management Act: Protected Areas Amendment (July 2009) | 🗶 | 🗶 | 🗶 | 🗶 |
| Marine Living Resources Act: Regulations: Management of boat-based whale watching and protection of turtles: Amendment (July 2009) | 🗶 | 🗶 | 🗶 | 🗶 |
| Marine Living Resources Act: Regulations: Management of white shark cage diving: Amendment (July 2009) | 🗶 | 🗶 | 🗶 | 🗶 |
| Marine Living Resources Act: Regulations: Management of boat-based whale watching and protection of turtles: Amendment (August 2009) | 🗶 | 🗶 | 🗶 | 🗶 |
| Marine Living Resources Act: Regulations: Management of white shark cage diving: Amendment (August 2009) | 🗶 | 🗶 | 🗶 | 🗶 |
| National Environmental Management Act: List of activities and competent authorities – amendment (October 2009) | 🗶 | 🗶 | 🗶 | 🗶 |
| National Environmental Management: Commencement of Integrated Coastal Management Act (December 2009) | 🗶 | ✓ | ✓ | ✓ |
| National Environmental Management: Protected Areas Act: Regulations for the proper administration of Knysna protected environment (December 2009) | 🗶 | 🗶 | ✓ | ✓ |
| National Environmental Management: Air Quality Act: National ambient air quality standards (December 2009) | 🗶 | ✓ | 🗶 | 🗶 |
| Marine Living Resources Act: Regulations amendment (February 2010) | 🗶 | 🗶 | 🗶 | 🗶 |
| National Environmental Management: Biodiversity Act: Convention on International Trade in Endangered Species (CITES) Regulations (March 2010) | 🗶 | 🗶 | ✓ | ✓ |
| National Environmental Management: Air Quality Act: Commencement of certain sections (March 2010) | 🗶 | 🗶 | 🗶 | 🗶 |
| National Environmental Management: Air Quality Act: List of activities which result in atmospheric emissions which have or may have significant detrimental effect on environment – commencement (March 2010) | 🗶 | ✓ | ✓ | ✓ |
| National Environmental Management Act: Environmental Management Framework Regulations (June 2010) | 🗶 | 🗶 | ✓ | 🗶 |
| National Environmental Management Act: Listing Notice 1: List of activities and competent authorities, (G 33306 - GoN 544) (June 2010) | 🗶 | 🗶 | ✓ | ✓ |
| National Environmental Management Act: Listing Notice 2: List of activities and competent authorities (June 2010) | ✓ | 🗶 | ✓ | ✓ |
| National Environmental Management Act: Listing Notice 3: List of activities and competent authorities (June 2010) | ✓ | 🗶 | ✓ | ✓ |
| National Environmental Management Act: Environmental Impact Assessment Regulations (June 2010) | 🗶 | 🗶 | ✓ | ✓ |
| National Environmental Management Act: Environmental Management Framework Regulations , (G 33306, - GoN 547) (June 2010) | 🗶 | 🗶 | ✓ | 🗶 |
| National Environmental Management Act: Model Air Quality Management By-law for adoption and adaptation by municipalities (July 2010) | ✓ | ✓ | ✓ | ✓ |
| National Environmental Management Act: Commencement of Environmental impact assessment regulations (July 2010) | 🗶 | 🗶 | 🗶 | 🗶 |
| National Environmental Management Act: Commencement of Environmental Management Framework regulations (July 2010) | 🗶 | 🗶 | 🗶 | 🗶 |
| National Environmental Management Act: Environmental Impact Assessment regulations and Listing notices amendments (July 2010) | 🗶 | 🗶 | 🗶 | 🗶 |
| National Environmental Management Act: Amendment of regulations for Control of use of vehicles in coastal zone (November 2010) | 🗶 | 🗶 | 🗶 | 🗶 |
| Marine Living Resources Act: Amendment of regulations published in notice R1111 of 2 September 1998, as amended (November 2010) | 🗶 | ✓ | 🗶 | ✓ |
| National Strategy for Sustainable Development and Action Plan (2011-2014) (NSSD 1) | ✓ | ✓ | ✓ | ✓ |
| DEA Supply Chain Management Policy (February 2011) | 🗶 | 🗶 | 🗶 | 🗶 |
| National Policy for the provision of basic refuse removal services to indigent households (June 2011) | 🗶 | ✓ | 🗶 | ✓ |
| National Environmental Management: Biodiversity Act: Threatened or Protected Species Amendment (July 2011) | 🗶 | 🗶 | 🗶 | 🗶 |
| The White Paper on National Climate Change Response (July/October 2011) | ✓ | ✓ | ✓ | ✓ |
| National Environment Management Act: Waste Act, 2008 (Act No 59 of 2008): Waste Classification and Management Regulation (G 35572 - GN 614) (August 2012) | 🗶 | ✓ | 🗶 | 🗶 |
| Act No.14 of 2003: National Environmental Management Laws Amendment, 2013 (July 2013) | ✓ | 🗶 | 🗶 | 🗶 |
| Act No.30 of 2013: National Environmental Management Laws Second Amendment Act, 2013 (December 2013) | 🗶 | 🗶 | 🗶 | ✓ |
| South African Weather Service (SAWS) Amendment Act, 2001 (Act 48 of 2013) | 🗶 | 🗶 | 🗶 | 🗶 |
| National Environmental Management: Waste Amendment Act (26 of 2014) (G 37714) (June 2014) | ✓ | ✓ | 🗶 | ✓ |
| National Environmental Management Laws Amendment Act (25 of 2014) (G 37713) (June 2014) | 🗶 | ✓ | 🗶 | ✓ |
| National Environmental Management Integrated Coastal Management Act, 2008 (Act No. 24 of 2008): Control of use of vehicles in the coastal area [G 37761 GN 496] (June 2014) | 🗶 | ✓ | ✓ | ✓ |
| National Environmental Management: Biodiversity Act 10 of 2004: Alien and Invasive Species Regulations September 2014 | 🗶 | ✓ | ✓ | ✓ |
| Marine Living Resources Act: Regulations: Amendment (December 2017) | 🗶 | 🗶 | 🗶 | 🗶 |
| Draft Climate Change Bill (June 2018) | 🗶 | ✓ | 🗶 | ✓ |
| South Africa’s National  Biodiversity Framework (2019-2024) | ✓ | ✓ | ✓ | ✓ |

# Supplementary Information 4

**KWAZULU-NATAL PROVINCIAL POLICIES ON AGRICULTURE**

**Table 4**: Review of KwaZulu-Natal agriculture-related policies and strategies

| **Name of policy** | **Human Health/Wellbeing** | **Land** | **Water** | **Environment/Climate change/Sustainable development** |
| --- | --- | --- | --- | --- |
| Strategic Plan 2015 - 2022 | 🗶 | ✓ | 🗶 | ✓ |
| Strategy for Agrarian Transformation in KZN (2015) | 🗶 | 🗶 | 🗶 | 🗶 |

# Supplementary Information 5

**KWAZULU-NATAL PROVINCIAL POLICIES ON HEALTH**

**Table 5:** Review of KwaZulu-Natal health-related policies and strategies

| **Name of policy** | **Agriculture/Food** | **Land** | **Water** | **Environment/Climate change/Sustainable development** |
| --- | --- | --- | --- | --- |
| KwaZulu-Natal Health Act (No.4 of 2000) | 🗶 | 🗶 | ✓ | ✓ |
| KwaZulu-Natal Health Act (2009) | 🗶 | 🗶 | 🗶 | ✓ |
| Strategic Plan (2015-2019) | 🗶 | 🗶 | ✓ | 🗶 |
| Health care risk waste management policy for KwaZulu-Natal province | 🗶 | 🗶 | 🗶 | ✓ |

# Supplementary Information 6

**KWAZULU-NATAL PROVINCIAL POLICIES ON ENVIRONMENT**

**Table 6:** Review of KwaZulu-Natal environment-related policies and strategies

| **Name of policy** | **Agriculture/Food** | **Human Health/Wellbeing** | **Land** | **Water** |
| --- | --- | --- | --- | --- |
| EDTEA Revised Strategic Plan 2014-2019 | 🗶 | 🗶 | 🗶 | 🗶 |
| EDTEA Revised Strategic Plan 2015-2020 | ✓ | 🗶 | ✓ | 🗶 |
| KZN Environmental Implementation Plan (EIP) | ✓ | ✓ | ✓ | ✓ |

# Supplementary Information 7

**KWAZULU-NATAL PROVINCIAL DEVELOPMENT STRATEGIES AND PLANS**

**Table 7:** Review of KwaZulu-Natal provincial development policies and strategies

| **Name of policy** | **Agriculture/Food** | **Human Health/Wellbeing** | **Land** | **Water** | **Environment/Climate change/Sustainable development** |
| --- | --- | --- | --- | --- | --- |
| KZN 2011 Provincial Growth and Development Plan | ✓ | ✓ | ✓ | ✓ | ✓ |
| KZN 2019 Provincial Growth and Development Plan | ✓ | ✓ | ✓ | ✓ | ✓ |
| KZN Legislature Strategic Plan 2014-2019 | \| 🗶 \| \| --- \| | ✓ | 🗶 | 🗶 | 🗶 |
| KZN Provincial Growth and Development Plan 2012-2030 | ✓ | ✓ | ✓ | ✓ | ✓ |
| KZN 2035 Provincial Growth and Development Strategy | ✓ | ✓ | ✓ | ✓ | ✓ |
| KwaZulu-Natal Poverty Eradication Master Plan (2014) | ✓ | ✓ | ✓ | ✓ | ✓ |
| Provincial Growth and Development Strategy and Plan Abridged Version October 2018 | ✓ | ✓ | ✓ | ✓ | ✓ |

**Table 8:** Policies that incorporate Agriculture (including land and water), Health, and Environment

| **POLICY** | **VISION/AIM/GOALS**  **OBJECTIVES**  **OF POLICY** | **SECTOR/GOVERNING DEPARTMENT** | **INCORPORATION OFAGRICULTURE, HEALTH AND ENVIRONMENT CONSIDERATIONS INTO POLICIES** | | |
| --- | --- | --- | --- | --- | --- |
|  |  |  | Agriculture | Health | Environment |
| National Environmental Health Policy (2013) | - To provide a national framework for the provision of environmental health services in the country, set out the vision for environmental health and influence health outcomes to ensure "A long and healthy life for all South Africans". | Health | The policy notes that climate change will have negative impacts on negatively on food production and water availability and human  beings are directly and indirectly exposed to these impacts e.g. by extreme weather related patterns  and by the negative impact on food, water, air, infrastructure, agriculture, ecosystems and livelihoods,  which can all lead to various environmental health issues.  DOH is concerned with food safety in respect of acceptable microbiological and chemical standards and  the fitness of all food for human consumption and optimal hygiene control throughout the food supply  chain from the point of origin to the point of consumption for protection of human health. |  | - The policy notes that human beings are directly and indirectly exposed to climate change, e.g. by extreme weather related patterns and by the negative impacts on food, water, air, infrastructure, agriculture, ecosystems and livelihoods, which can all lead to various environmental health issues - The policy notes that environmental health interventions should respond to the differing needs of women, men, children and the elderly. This must apply specifically on the role of women as main users of food, water and sanitation. |
| National Climate Change and Health Adaptation Plan (2014-2019) | - To provide a broad framework for health sector action towards implementation of the National Climate Change Response Policy (NCCRP) - To effectively manage inevitable climate change impacts on health through interventions that build and sustain South Africa’s socio-economic and environmental resilience and emergency response capacity - Describe the environmental and health contexts for the proposals contained in this plan - Outline a broad programme of activities to be undertaken or spearheaded by the South African health sector, giving specific examples - Indicate the potential partners, time frames and financial implications | Health | - The plan highlights that while South Africa is known to be food secure, nationally, the distribution, access and utilisation of food remains a challenge, resulting in poor health outcomes. |  | - The plan notes that linkages exist between the health and the environment, and managing environmental risks related to climate change and management of natural and human disasters are amongst the top priority health and environment issues in the country |
| White Paper on Agriculture (1995) | - To ensure equitable access to agriculture and promote the contribution of agriculture to the development of all communities, society at large and the national economy, in order to enhance income, food security, employment and quality of life in a sustainable manner. | Agriculture |  | The Paper notes that agricultural productivity has an impact on household food security and health. Poor productivity can lead to malnutrition, especially in rural areas | - The paper notes that that certain agricultural activities are causing environmental damage, in particular, in rural areas where environmental damage in rural areas is on the increase due to incorrect irrigation methods, the excessive use of pesticides and fertilisers and the pollution of surface and groundwater by industries, power stations and mines. - Rural biodiversity is also threatened by the spread of exotic and invasive plant species,   the increasing use of land for forestry and the destruction of indigenous forests by agriculture. |
| Water Services Act (No. 108 of 1997) | - To provide for the rights of access to basic water supply and basic sanitation - To provide for the setting of national standards and of norms and standards for tariffs - To provide for water services development plans; to provide a regulatory framework for water services institutions and water services intermediaries - To provide for the establishment and disestablishment of water boards and water services committees and their powers and duties; to provide for the monitoring of water services and intervention by the Minister or by the relevant Province - To provide for financial assistance to water services institutions - To provide for certain general powers of the Minister - To provide for the gathering of information in a national information system and the distribution of that information - To repeal   certain laws; and   - To provide for matters connected therewith | Agriculture |  | The Act states that the right of access to basic and sufficient water supply and the right to basic sanitation are important for human  health and well-being | The Act notes that water services may have an impact on the environment, therefore, it is necessary to comply with the country’s environmental policies |
| Pesticide Management Policy for South Africa (2010) | - To improve legislative framework to ensure that South Africans are better protected from health and environmental risks posed by pesticides - To encourage the development and use of alternative products and techniques and reduce dependence on chemical plant protection products - To integrate relevant international agreements and initiatives from other government departments - Increased transparency. access to information and improve public participation in the registration of pesticides | Agriculture |  | - The Policy recognises that pesticide use can have adverse effects on human health and, therefore, calls for the effective and efficient management of pesticides - The Policy adds that the responsibility for the enforcement of pesticide regulation will be shared among the Department of Health, Trade and Industry, Finance (Custom and Excise) Labour, Water Affairs, Environmental Affairs, with the Department of Agriculture, Forestry and Fisheries (DAFF) having the statutory responsibility - The policy states that human health, environmental quality and economic development depend on effective systems that enable South Africans to manage and use pesticides safely and sustainably. | - The Policy recognises that pesticide use can have adverse effects on human health and, therefore, calls for the effective and efficient management of pesticides - The Policy adds that the responsibility for the enforcement of pesticide regulation will be shared among the Department of Health, Trade and Industry, Finance (Custom and Excise) Labour, Water Affairs, Environmental Affairs, with the Department of Agriculture, Forestry and Fisheries (DAFF) having the statutory responsibility |
| Integrated growth and development plan (IGDP) for agriculture, forestry and fisheries (2012) | To promote equitable, productive, competitive, profitable and sustainable  agriculture, forestry and fisheries sectors, growing to the benefit of all South Africans | Agriculture |  | - The Plan seeks to promote responsible and sustainable use of the living aquatic resources and aquatic ecosystems of interest to State parties in order to promote and enhance food security and human health | - The Plan notes the fisheries sector contributes to environmental degradation and one of the biggest challenges that the sector faces is that of balancing the high demand for access to marine living resources as a means of household income and subsistence, with the need to ensure the environmental sustainability of resources - The Plan states that aquaculture can also have a negative impact on the environment, most notably on water quality and genetic integrity of wild stocks, alien species and disease - The Plan states that climate change will have an impact on all three sectors – agriculture, forestry and fisheries- mitigation and adaptation strategies are of real concern |
| Agricultural Policy Action Plan (APAP) (2014-2019) | - The APAP seeks to translate the high-level responses offered in the Integrated growth and development plan (IGDP) for agriculture, forestry and fisheries (2012) into tangible, concrete steps - The APAP is planned over a five-year period and will be updated on an annual basis - Aligning itself with the New Growth Path (NGP), the National Development Plan (NDP) and Industrial Policy Action Plan (IPAP), APAP seeks to assist in the achievement of Outcome 4, Decent Employment through Inclusive Growth, and that of Outcome 7, Comprehensive Rural Development and Food Security | Agriculture |  | - The Plan reports that climate change is likely to have a severe impact on hunger and malnutrition levels in some of the provinces in the country, namely, the Eastern Cape, Free State and North West. | - The Plan notes that agriculture has an impact on the environment - The Plan adds that the agricultural sector is one of the contributors of greenhouse gases (GHG) emissions globally which contribute to the changing climate - The agricultural sector is equally one of the sectors that are vulnerable to varying climatic conditions resulting from the changing climate. This leads to the declining production capacity of farming systems, affecting food security - Unsustainable land use practices, the current a state of natural resources degradation coupled with the varying climatic conditions undermine production |
| National Policy on Food and Nutrition Security (2014) | To ensure the  accessibility and affordability of safe and nutritious food at national and  availability,  household levels | Agriculture |  | - The Policy notes that access to good quality, safe and nutritious food is essential for good health | - The Policy notes climate change and environmental damage threaten domestic food production and household food security and that food and nutrition security requires well-managed inter-sectoral co-ordination, and the genuine integration of existing policies and programmes in health, education, and environmental protection, as well as in agrarian reform and agricultural development. |
| Draft Climate Smart Agriculture Strategic Framework (2018) | To promote effective adaptation responses and increase adaptive capacity in order to reduce vulnerability and increase overall resilience of South Africa’s Agriculture, Forestry and Fisheries (AFF) systems, including their socio-economic and institutional characteristics | Agriculture |  | - The Framework notes that climate change can have serious effects on South Africa’s low lying areas, infrastructure, socio-economic activities, human health, water resources and food security | - The framework acknowledges that agriculture contributes to greenhouse gases (GHG) emissions and these contribute to climate variability and change. Climate change, in turn, poses risks to various key sectors of the South African economy, *viz*., water, agriculture and forestry. - The Framework adds that climate change is currently negatively impacting on agriculture production both in aquaculture, plant and animal farming- which will inherently impact negatively on the sustainability of the agriculture sector and its ability to provide food and employment opportunities |
| Draft White Paper on Conservation and Sustainable Use of Biodiversity (1997) | VISION: A prosperous, environmentally conscious nation, whose people are in harmonious co-existence  with the natural environment, and which derives lasting benefits from the conservation and  sustainable use of its rich biological diversity  In addition, the Paper states that because of the cross-sectoral nature of biodiversity, several other national government  departments will play a vital role in the implementation of this policy. These include the Departments of Agriculture; Land Affairs; Water Affairs and Forestry; Trade and Industry; Foreign Affairs; Health; Transport; Housing; Welfare and Population Development; Arts,  Culture, Science and Technology; Finance; as well as the South African National Defence Force.  Of crucial importance will be their commitment to cooperating with one another, and to  developing sectoral-specific plans and budgets to reflect how biodiversity considerations will  be incorporated into the activities of departments. | Environment | - The Paper notes that the agriculture and environment sectors are linked, stating that the agriculture sector directly uses ecosystems to provide adequate natural grazing for livestock, and can similarly be identified as being directly dependent upon indigenous biological resources, and the adequate renewal thereof. - The Paper also notes that agricultural activity has been changing South African ecosystems as a substantial proportion of natural habitat has been transformed - In addition to habitat loss and degradation, the overexploitation of certain species, the introduction of exotic species, and the pollution or toxification of the soil, water and atmosphere have had major effects on South Africa's terrestrial, freshwater and marine biodiversity - The Paper states that there is need for coordination and cooperation because biodiversity transcends political, institutional and social boundaries. - An enabling framework needs to be provided for the future coordination and cooperation of biodiversity-related activities in South Africa, in the southern African sub-region, and globally - Coordination will also be ensured between other plans, programmes and policies which have implications for the conservation of biodiversity and use of biological resources. - The Paper also calls for the conservation and sustainable use of biodiversity to be integrated strategically at all levels into national, provincial, local and sectoral planning, programme, and policy efforts (e.g. forestry, agriculture, fisheries, land reform, industry, education, health, mining. etc.) in order to implement the goals and objectives of the policy effectively | - The Paper stated that there are links between biodiversity conservation and community health and welfare - The Paper calls for the conservation and sustainable use of biodiversity to be integrated strategically at all levels into national, provincial, local and sectoral planning, programme, and policy efforts (e.g. forestry, agriculture, fisheries, land reform, industry, education, health, mining. etc.) in order to implement the goals and objectives of the policy effectively |  |
| White Paper on Environmental Management Policy (1998) | VISION: to unite the people of South Africa in working towards a society where all people have sufficient food, clean air and water, decent  homes and green spaces in their neighbourhoods enabling them to live in spiritual, cultural and physical harmony with their natural surroundings | Environment | - The Paper notes that there is a link between agriculture and the environment. Therefore, it seeks to ensure the sustainable use of natural resources in the agricultural economy and sustainable forest development; to promote and encourage sustainable low input farming systems; to regulate the use of toxic and hazardous chemicals in agriculture to protect human health and the environment | The Paper states that it is necessary to ensure environmental health as it is important for human health |  |
| White Paper on Integrated Pollution and Waste Management (2000) | VISION: To develop, implement and maintain an integrated pollution and waste  management system which contributes to sustainable development and a  measurable improvement in the quality of life, by harnessing the energy and  commitment of all South Africans for the effective prevention, minimisation  and control of pollution and wate. | Environment | - The Paper notes that agriculture is a source of air pollution and the land pollution is supposed to be regulated by the Department of Agriculture, the Department of   Water Affairs and Forestry, the Department of Minerals and Energy and other  pollution control authorities   - Furthermore, the Paper states that the Department of Agriculture is supposed to develop the necessary regulations and guidelines for all   agricultural wastes, in consultation with the Department of Environmental Affairs and Tourism (DEAT) | - The Paper notes that environment pollution such as air and land/soil pollution can cause human health problems, therefore, it is necessary to manage, prevent, reduce and control all identified forms of pollution |  |
| National Biodiversity Strategy and Action Plan (2005) | Conserve and manage terrestrial and aquatic biodiversity to ensure  sustainable and equitable benefits to the people of South Africa,  now and in the future | Environment | - The Plan notes that agriculture is one of the sectors that has an impact on biodiversity as it transforms land and uses large amounts of water - The Plan adds that agriculture has major negative impacts on biodiversity, especially where natural vegetation is completely cleared for crops, therefore, the sector needs to factor biodiversity considerations into its policies, plans and programmes - The Plan also notes that the biodiversity conservation sector in South Africa is well established. The Department of Environmental Affairs and Tourism (DEAT) is the primary custodian of the environment, but this responsibility is shared. Biodiversity is also an important function of other national departments such as Department of Water Affairs and Forestry (DWAF) and the Department of Agriculture (DoA), and a number of other public and private (civic) institutions | - The Plan notes that any threats to ecosystems, habitats or other species may result in economic or environmental harm or harm to human health. An example of such a threat is invasive species - The Plan also notes that poor waste management and inadequate pollution control can have a serious impact on both natural ecosystems and human health |  |
| National Framework for Sustainable Development (July 2008) | VISION: South Africa aspires to be a sustainable, economically prosperous and self-reliant nation state that  safeguards its democracy by meeting the fundamental human needs of its people, by managing  its limited ecological resources responsibly for current and future generations, and by advancing  efficient and effective integrated planning and governance through national, regional and global  collaboration. | Environment | - The Framework notes that agricultural production, including access to food, in many African countries and regions is projected to be severely compromised by climate variability and change. The area suitable for agriculture, the length of growing seasons and yield potential, particularly along the margins of semi-arid and arid areas, are expected to decrease. This would further adversely affect food security and exacerbate malnutrition in the continent. - The Framework states that to minimise further biodiversity loss, it will be necessary to foster working relations between the biodiversity protection agencies and production sectors such as major land users (including agriculture, infrastructure and property development, forestry, fisheries and mining) in order to develop and implement sector specific initiatives to prevent further loss and degradation of natural habitat in threatened ecosystems. | - The Framework notes that environmental pollution affects human health and health costs can be reduced by cleaning up air pollution, eliminating waste altogether via recycling, replacing high energy lighting systems with low energy and cheaper lighting, and reducing congestion by replacing private passenger transport via mass public transit systems |  |
| White Paper on National Climate Change Response (NCCR) (2011) | - To effectively manage inevitable climate change impacts through interventions that build and sustain South Africa’s social, economic and environmental resilience and emergency response capacity - To make a fair contribution to the global effort to stabilise   GHG concentrations in the atmosphere at a level that  avoids dangerous anthropogenic interference with  the climate system within a timeframe that enables  economic, social and environmental development to  proceed in a sustainable manner. | Environment | - The Paper highlights that ecosystems provide important services to society, including food - The Paper notes that land-based human activities, such as forest clearing and unsustainable agricultural practices, are not only increasing GHG emissions from these sources, but are also reducing the earth’s natural ability to absorb GHGs - The Paper adds that the evidence that current global warming is due to human activities associated with industrialisation and modern agriculture is overwhelming - The Paper notes that climate change poses significant additional risks for water security, which in turn has knock-on effects on those sectors highly reliant on water such as agriculture, electricity generation as well as some mining and industrial activities - The Paper states that it is necessary to integrate climate change considerations in the short-, medium- and long-term water planning processes across relevant sectors such as agriculture, industry, economic development, health, science and technology - The Paper adds that it is also necessary to integrate agriculture and forestry into climate resilient rural development planning to address job creation, food security and livelihoods with a particular emphasis on building climate resilience through leveraging synergies between adaptation and mitigation | - The Paper highlights the strong links between weather and disease - The Paper also highlights the impact of climate change on health, noting that the negative impacts of climate change on the socio-economic standing of the most vulnerable communities, and the consequences in terms of food security and the nutritional status of individuals within these communities threatens to further undermine their resistance to diseases such as HIV/AIDS and tuberculosis - The Paper states that integrating climate change considerations in the - short-, medium- and long-term water planning - processes across relevant sectors such as agriculture, - industry, economic development, health, science and - technology. |  |
| National Strategy for Sustainable Development and Action Plan (2011-2014) (NSSD 1) | South Africa aspires to be a sustainable, economically prosperous and self-reliant nation that safeguards its democracy by meeting the fundamental human needs of its people, by managing its limited ecological resources responsibly for current and future generations, and by advancing efficient and effective integrated planning and governance through national, regional and global collaboration | Environment | - The Plan notes that functioning ecosystems produce goods- natural products, such as water, timber, flowers, food and medicines - The Plan notes that climate change has an impact on the agriculture sector, and food security (by reducing crop yields and altering rainfall patterns) and there is a need to strengthen the sector to be more resilient and also have the ability to adapt | - The Plan notes that climate change has an impact on the health sector and there is a need to strengthen the sector to be more resilient and also have the ability to adapt - The Paper adds that pollution also poses a threat to water scarcity and will have a serious impact on people’s health |  |
| South Africa’s National  Biodiversity Framework (2019-2024) | Conserve, manage and sustainably use biodiversity to ensure  benefits to the people of South Africa, now and in the future | Environment | - The Framework notes that there is a link between biodiversity and agriculture, stating that while the sector’s core work is not biodiversity conservation, its business impacts on the status of biodiversity and ecosystems, or depends on biodiversity assets, ecosystem services and ecological infrastructure - The Framework states that many decisions affecting biodiversity are taken outside of the biodiversity or environmental sector (for example, agriculture, mining, energy and urban development), which makes it important to adopt a framework for integrated, policy-aligned decision-making | - The Framework notes that good management of biodiversity assets contributes to the economy, rural development and job creation and social wellbeing |  |
| **KZN** |  |  |  |  |  |
| KZN Environmental Implementation Plan (EIP) | - To co-ordinate and harmonise the environmental policies, plans, programmes and decisions of the various national departments that exercise functions that may affect the environment or are entrusted with powers and duties aimed at the achievement, promotion, and protection of a sustainable environment, and of provincial and local spheres of government, in order to:  1. minimise the duplication of procedures and functions; and 2. promote consistency in the exercise of functions that may affect the environment;  - give effect to the principle of co-operative government in chapter 3 of the Constitution - secure the protection of the environment across the country as a whole - prevent unreasonable actions by provinces in respect of the environment that are prejudicial to the economic or health interests of other provinces or the country as a whole; and - enable the Minister to monitor the achievement, promotion, and protection of a sustainable environment | KZN Environment | - The Plan states that destructive agricultural practices such as overuse of fertilizers and overgrazing lead to land degradation - The Plan notes that agriculture is one of the major sources of air pollution in KZN - The Plan states that in South Africa, while the lead agent for environmental governance is the National Department of Environmental Affairs (DEA), environmental functions are spread across a range of ministries, for instance the Department of Water Affairs (DWA) with its mandate for water security, and the Department of Agriculture, Forestry and Fisheries (DAFF) with its mandate to conserve agricultural resources | - The Plan states that unmanaged disposal of solid waste   causes pollution and degradation of the natural environment, posing a health risk to humans |  |
